# Inhibition of ferroptosis protects House Ear Institute‐Organ of Corti 1 cells and cochlear hair cells from cisplatin‐induced ototoxicity

**DOI:** 10.1111/jcmm.15839

**Published:** 2020-09-14

**Authors:** Honglin Mei, Liping Zhao, Wen Li, Zhiwei Zheng, Dongmei Tang, Xiaoling Lu, Yingzi He

**Affiliations:** ^1^ ENT Institute and Department of Otorhinolaryngology Eye & ENT Hospital Fudan University Shanghai China; ^2^ NHC Key Laboratory of Hearing Medicine (Fudan University) Shanghai China

**Keywords:** cisplatin, ferroptosis, mitochondrial function, ototoxicity, reactive oxygen species

## Abstract

Ferroptosis is a recently recognized form of non‐apoptotic cell death caused by an iron‐dependent accumulation of lipid hydroperoxides, which plays important roles in a wide spectrum of pathological conditions. The present study was aimed to investigate the impact of ferroptosis on cisplatin‐induced sensory hair cell damage. Cell viability was determined by Cell Counting Kit‐8 and lactase dehydrogenase assays. The reactive oxygen species (ROS) levels were evaluated by 2,7‐Dichlorodi‐hydrofluorescein diacetate (DCFH‐DA) and MitoSox‐Red staining. Mitochondrial membrane potential (MMP) was measured by tetramethylrhodamine methyl ester (TMRM) staining. Lipid peroxidation, intracellular and mitochondrial iron were detected by Liperfluo, C11‐BODIPY^581/591^, FerroOrange and Mito‐FerroGreen, respectively. We found that cisplatin treatment not only markedly augmented ROS accumulation, decreased the MMP, but increased lipid peroxidation and iron accumulation in House Ear Institute‐Organ of Corti 1 (HEI‐OC1) cells. Of note, treatment with the specific ferroptosis inhibitor ferrostatin‐1 could effectively abrogate the cisplatin‐induced toxicity and subsequent cell death. Specifically, the improvement of mitochondrial functions is important mechanisms for protective action of ferroptosis inhibitor against cisplatin‐induced damages in HEI‐OC1 cells. Moreover, inhibition of ferroptosis significantly protected murine cochlear hair cells against cisplatin damage. In addition, treatment murine cochlear hair cells with ferroptosis inducer, RSL3, significantly exacerbated cisplatin‐induced damage, which could be alleviated by ROS inhibitor N‐acetyl‐L‐cysteine. Collectively, our study indicated that ferroptosis inhibition could alleviate the cisplatin‐induced ototoxicity via inactivation of lipid peroxide radical and improvement of mitochondrial function in hair cells.

## 1 INTRODUCTION

Hearing loss is a very common sensory disorder in humans and is mainly attributable to hair cell damage caused by ototoxic pharmaceutical agents, excessive noise, ageing and genetic disorders. Cisplatin is one of the largest classes of valuable clinical chemotherapeutic agents against various malnancies.^1^, ^2^ However, the ototoxicity induced by cisplatin is one of the most serious adverse effects of cisplatin administration, which results in irreversible, progressive, bilateral and accumulative hearing impairment, thereby limiting the clinical use of cisplatin.^3^, ^4^ Numerous studies have shown that cochlear hair cells and spiral ganglion neurons are major targets, and exposure of these cells to cisplatin induces a significant cell death.^5^, ^6^, ^7^ Though it has been shown that the cisplatin‐induced ototoxicity is associated with the reactive oxygen (ROS)/nitrogen species accumulation, mitochondrial dysfunction and caspase activation in auditory organs,^8^, ^9^ exact mechanisms involved in cisplatin damage have still not been fully clear, thereby better understanding the underlying mechanisms of cisplatin‐induced ototoxicity are crucial for seeking more effective therapies for preventing hearing loss.

Ferroptosis is a recently recognized type of non‐apoptotic cell death that is characterized by the overload of free ferrous ion and the accumulation of lipid‐based ROS; it is biochemically and morphologically distinct from apoptosis, necroptosis and autophagy.^10^ The accumulation of lipid peroxides is mainly caused by the reduced activity of glutathione peroxidase 4 (GPX4), which is a unique intracellular antioxidant enzyme that suppresses lipid peroxidation generation in the cell membrane.^11^ Another key regulator in ferroptosis is the cystine/glutamate antiporter system $x_{c}^{‐}$ (xCT), which exchanges extracellular cystine for intracellular glutamate.^12^ There are increasing studies showing that ferroptosis inducers, such as RSL3, inhibiting the function of GPX4,^13^ and erastin, inhibiting xCT,^14^, ^15^ have been confirmed to enhance sensitivity of drug‐resistant cancer cells to chemotherapeutic drugs such as cisplatin and temozolomide thereby exhibiting anticancer effects. Several inhibitors of ferroptosis have recently been identified, including liproxstatin‐1,^16^ ferrostatin‐1 (FER‐1)^17^ and the iron chelator deferoxamine (DFO). Inhibition of accumulation of lipid peroxidation that inhibits ferroptosis could present highly promising way to treat pathological conditions by protecting from the cell loss in the brain, liver, kidney and other tissues.^16^, ^18^, ^19^ In vivo studies with ferroptosis inhibitors highlighted the importance of inhibition of this death pathway in mitigating cell damage.^16^, ^18^ To date, there has been no study with regard to ferroptosis involvement in cisplatin‐induced ototoxicity.

In this study, we investigated the involvement of ferroptosis in cisplatin‐induced hair cell damage, and the potential protective effect of ferroptosis inhibition in alleviating the impairment of hair cells induced by cisplatin administration in both auditory House Ear Institute‐Organ of Corti 1 (HEI‐OC1) cells and murine cochleae. Our results showed that inhibition of ferroptosis with FER‐1 significantly attenuated cisplatin‐induced hair cell damage by preserving mitochondrial function, suggesting that inhibition of ferroptosis might be a novel therapeutic target for future hearing loss treatment.

## 2 MATERIALS AND METHODS

### 2.1 HEI‐OC1 cell culture

House Ear Institute‐Organ of Corti 1 cells were cultured in high‐glucose DMEM (Gibco BRL, Gaithersburg, MD, USA) supplemented with 5% volume of foetal bovine serum (Gibco BRL) without antibiotics in acceptable conditions (5% CO_2_, 33°C).

### 2.2 Postnatal cochlear explants culture

Postnatal day (P) 2 C57BL/6 mice were sacrificed and soaked in 75% alcohol, and the cochleae tissues were carefully dissected using scissors and placed in cooled PBS. The cochlea was then stuck to a glass of coverslip coated with Cell‐Tak (BD Biosciences, Franklin Lakes, NJ, USA). Finally, DMEM/F12 medium supplemented with N2/B27 (Invitrogen) and ampicillin was added, and the cochleae tissues were cultured in a 5% CO_2_/95% air atmosphere at 37°C overnight prior to each treatment. All experimental procedures on animals in this study were conducted in accordance with the laboratory animals care guidelines and approved by the Institutional Animal Care and Use Committee of Fudan University.

### 2.3 Drug treatments

RSL3, FER‐1, DFO, liproxstatin‐1 (Lip‐1) and Z‐VAD‐FMK were purchased from Selleck (Chemicals, Houston, TX). Cisplatin and N‐acetyl‐L‐cysteine (NAC) were purchased from Sigma‐Aldrich (Saint Louis, USA). RSL3, FER‐1, DFO, Lip‐1, Z‐VAD‐FMK and NAC were initially dissolved in dimethylsulfoxide (DMSO) and applied at final concentrations (1, 2, 3 and 5 μmol/L with RSL3; 0.5, 1, 2, 5, 10, 20, 30 and 40 μmol/L with FER‐1; 5, 10, 20, 40, 60 and 80 μmol/L with DFO; 0.5, 1, 2, 5, 10 and 20 μmol/L with Lip‐1; 1, 2, 5, 10, 20 and 40 μmol/L with Z‐VAD‐FMK; 5 mmol/L with NAC). Cisplatin was supplied as a 1 mmol/L stock solution in PBS and diluted in culture medium. Final cisplatin concentrations ranged from 10 to 40 μmol/L.

### 2.4 Cell viability quantification

Cell Counting Kit‐8 (CCK‐8) (Sigma, Saint Louis, USA) reagent was used to examine cell viability according to the manufacturer's instructions. In brief, the cultured HEI‐OC1 cells were seeded at the density of 5000 cells/well in 96‐well plates in three replicates and allowed to attach overnight. At the end of different treatments, 10 μL per well CCK‐8 reagent was added to each well in incubation for 2 hours. Absorbance at 450 nm was detected by a plate reader (BioRad, Hercules, CA, USA).

### 2.5 Lactase dehydrogenase release assay

The extent of cellular injury was determined by lactase dehydrogenase (LDH) leakage using LDH cytotoxicity detection kit (Dojindo Laboratory, Kumamoto, Japan). Briefly, HEI‐OC1 cells were seeded at the density of 5000 cells/well in 96‐well plates. According to the manufacturer's instruction, at the end of different treatments, 100 μL of fresh reaction mixture was added to each well and incubated for 30 minutes, the absorbance at 490 nm was determined using a microplate reader (BioRad).

### 2.6 ROS measurement

The level of intracellular and mitochondrial ROS was respectively evaluated by the uptake of DCFH‐DA (Molecular Probes, Life Technologies, Invitrogen, Eugene, OR, USA) and MitoSox‐Red (Molecular Probes, Life Technologies). To visualize the ROS production, HEI‐OC1 cells were seeded in 8‐well ibidi plates with 15 000 cells/well and treated with the designate conditions. After the indicated time of treatment, cells were double stained with 50 μmol/L DCFH‐DA and 5 μmol/L MitoSox‐Red. After 30 minutes in the dark, the cells were stained with Hoechst nuclear stain (Dojindo) for 10 minutes. Images were acquired using a Leica SP8 confocal laser scanning microscope (Leica Microsystems, Wetzlar, Germany) equipped with a Plan Apochromat 60 × 1.40 NA oil immersion objective, and optical sections were collected every 1.5 μm to generate z‐stacks. Each image represented the maximum projection of all slices along the z‐stack. For evaluation of ROS production, HEI‐OC1 cells were seeded in 24‐well plates with 50 000 cells/well. After different treatment, cells were stained with 50 μmol/L DCFH‐DA or 5 μmol/L MitoSox‐Red for 30 minutes in the dark at 37°C, respectively. After collecting and washing with PBS, cells were re‐suspended in PBS and green or red fluorescence was detected by FACS analysis (FACScan; BD Biosciences, San Jose, CA, USA). Data were collected from at least 20 000 cells. DCFH‐DA fluorescence intensity in cochleae was analysed over a stretch of 200 µm of the middle turn. Each experiment was repeated three times, and exact numbers of cochlear explants (n) are indicated in the legends. The fluorescence intensity was measured by ImageJ (National Institutes of Health). The mean fluorescent intensity of each group was normalized to that of the control group.

### 2.7 Lipid peroxides measurement

To visualize the lipid ROS, cells were seeded in 8‐well ibidi plates and treated with the designate conditions. After different treatments, cells were stained with 5 μmol/L Liperfluo (Dojindo) or 2 μmol/L C11‐BODIPY^581/591^ probe (Molecular Probes Inc., Eugene, Oregon, USA) in accordance with the manufacturer's instructions. After 30 minutes at 37°C in the dark, the cells were stained with Hoechst nuclear stain (Dojindo) for 10 minutes. Subsequently, cells were washed with PBS and observed using a Leica SP8 confocal laser scanning microscope (Leica Microsystems) equipped with a Plan Apochromat 60 × 1.40 NA oil immersion objective, and slices were collected every 1.5 μm to generate z‐stacks. Each image represented the maximum projection of all slices along the z‐stack. Analysis of Liperfluo was performed by measuring the intensity of fluorescence of at least 180 cells for each treatment condition. These cells were from at least nine randomly selected regions of interest across three independent experiments. The fluorescence intensity was measured by ImageJ (National Institutes of Health). The mean fluorescent intensity of each group was normalized to that of the control group. Analysis of C11‐BODIPY^581/591^ fluorescence was performed by FACS analysis (FACScan; BD Biosciences, San Jose, CA, USA) and then evaluated with the FlowJo 7.6 software. Data were collected from at least 20 000 cells.

### 2.8 Mitochondrial membrane potential measurement

Mitochondrial membrane potential (MMP) was estimated by TMRM (Molecular Probes Inc.) staining. HEI‐OC1 cells were seeded in 8‐well ibidi plates with 15 000 cells/well and treated with the designate conditions for the indicated amount of time before staining with TMRM at a final concentration of 200 nmol/L for 30 minutes at 37°C. Images were acquired using a Leica SP8 confocal laser scanning microscope (Leica Microsystems) equipped with a 60 × 1.40 NA oil immersion objective. Slices were collected every 1.5 μm to generate z‐stacks, and each image represented the maximum projection of all slices along the z‐stack. For evaluation of the changes in the MMP, HEI‐OC1 cells were seeded in 24‐well plates with 50 000 cells/well and stained with TMRM for 30 minutes at 37°C. After collecting and washing with PBS, cells were re‐suspended in PBS and TMRM fluorescence was assessed by FACS analysis (FACScan; BD Biosciences, San Jose, CA, USA). Data were collected from at least 20 000 cells.

### 2.9 MitoTracker Red CMXRos staining

For analysis of mitochondrial morphology, HEI‐OC1 cells were seeded at 15 000 cells/well in 8‐well ibidi plates and treated with the designate conditions for the indicated amount of time. Afterwards, cells were stained with 200 nmol/L MitoTracker Red CMXRos (Molecular Probes Inc.) for 15 minutes at 37°C followed by incubation with Hoechst nuclear stain (Dojindo) for 10 minutes shielded from light. Subsequently, cells were washed with PBS and then randomly captured by a Leica SP8 confocal laser scanning microscope (Leica Microsystems) with a Plan Apochromat 60 × 1.40 NA oil immersion objective. Slices were collected every 1.5 μm to generate z‐stacks, and each image represented the maximum projection of all slices along the z‐stack. Images were determined across nine randomly selected fields from three independent experiments.

### 2.10 Fe^2+^ detection

To detect intracellular and mitochondrial Fe^2+^, FerroOrange and Mito‐FerroGreen (Dojindo) were used according to the manufacturer's protocol. HEI‐OC1 cells and cochleae were treated with cisplatin ± FER‐1 or RSL3 for the indicated amount of time and stained with a final concentration of 1 μmol/L FerroOrange or 5 μmol/L Mito‐FerroGreen for 30 minutes at 37°C. Images were acquired using a Leica SP8 confocal laser scanning microscope (Leica Microsystems) equipped with a Plan Apochromat 60 × 1.40 NA oil immersion objective. Slices were collected every 1.5 μm to generate z‐stacks, and each image represented the maximum projection of all slices along the z‐stack. Analysis of Fe^2+^ in HEI‐OC1 cells was performed by measuring the intensity of fluorescence of at least 180 cells for each treatment condition. These cells were from eight randomly selected regions across three independent experiments. Fe^2+^ fluorescence intensity in cochleae was analysed over a stretch of 200 µm of the middle turn. For each experimental condition, at least three cochleae were analysed, and each experiment was repeated three times. Exact numbers of cochlear explants (n) are indicated in the legends. The fluorescence intensity was measured by ImageJ (National Institutes of Health). The mean fluorescent intensity of each group was normalized to that of the control group.

### 2.11 Measurement of GSH/GSSG ratio

The intracellular total glutathione (GSH) and glutathione disulphide (GSSG) levels were detected by GSH Kit (Dojindo) according to the manufacturers' protocols. The relative levels were analysed on the microplate reader (BioRad).

### 2.12 Propidium iodide staining

Cell death was determined using Hoechst 33342/propidium iodide (PI) staining (Dojindo). After HEI‐OC1 cells were seeded in 8‐well ibidi plates and treated with different conditions, the cells were stained with PI (5 μg/mL) and Hoechst 33 342 (5 μg/mL). After 10 minutes in the dark, the cells were then examined using a Leica SP8 confocal fluorescence microscope (Leica Microsystems) equipped with a 60 × 1.40 NA oil immersion objective. Slices were collected every 1.5 μm to generate z‐stacks, and each image represented the maximum projection of all slices along the z‐stack. For evaluation, HEI‐OC1 cells were seeded in 24‐well plates with 50 000 cells/well. After different treatment, cells were stained with PI (5 μg/mL) for 10 minutes in the dark. After collecting and washing with PBS, cells were re‐suspended in PBS and red fluorescence was detected by FACS analysis (FACScan; BD Biosciences, San Jose, CA, USA). Data were collected from at least 20 000 cells.

### 2.13 Immunofluorescence

The cochleae fixed in 4% paraformaldehyde were rinsed three times with PBS and permeabilized for 30 minutes with 1% Triton X‐100 in PBS (PBST), blocked with 10% donkey serum in PBST for 1 hour followed by incubation with the primary antibodies overnight. The primary antibodies were anti‐myosin 7a antibody (Proteus Biosciences, Ramona, CA, USA), anti‐4‐hydroxynonenal (4‐HNE) antibody (Abcam, Cambridge, MA, USA), anti‐parvalbumin antibody (Abcam) and anti‐cleaved caspase‐3 antibody (Cell Signaling Technology, Inc, Danvers, MA, USA). Then, they were washed three times with PBS and incubated with secondary fluorescent antibodies for 1 hour at 37°C in the dark and stained with 4′,6‐diamidino‐2‐phenylindole (DAPI) (Sigma‐Aldrich) to label nuclei. The specimens then were visualized with a Leica SP8 confocal fluorescence microscope (Leica Microsystems). A Plan Apochromat objective (60 × 1.40 NA oil) was used with 0.75 × zoom setting, and optical sections were collected every 2 μm to generate z‐stacks. Images were assembled, viewed and processed using the Leica Application Suite Advanced Fluorescence software (Leica Microsystems) and Photoshop CS6 (Adobe Systems Inc., San Jose, California, USA). 4‐HNE fluorescence intensity was analysed over a stretch of 200 µm of the middle turn of cochleae. Each experiment was repeated three times, and exact numbers of cochlear explants (n) are indicated in the legends. The intensity of fluorescence was measured by ImageJ (National Institutes of Health). The mean fluorescent intensity of each group was normalized to that of the control group.

### 2.14 Western blot analysis

Cochleae were lysed with cold radio‐immunoprecipitation assay (RIPA) lysis buffer plus PMSF. The lysed cells were centrifuged at 21 000 *g* for 10 minutes at 4°C. The supernatant was collected, and protein concentrations were measured using a bicinchoninic acid (BCA) protein kit (Beyotime Institute Biotechnology, Nanjing, China). Equal amounts of each protein sample were separated via 12% SDS‐PAGE and transferred to positively charged nylon (PVDF) membranes (Immobilon‐P; Millipore, Schaffhausen, Switzerland). After blocking with skim milk for 1 hour in TBST (25 mmol/L Tris [pH 7.6], 138 mmol/L NaCl and 0.05% Tween‐20), the membranes were probed with anti‐GPX4 (1:1000; Abcam), anti‐cleaved‐poly ADP‐ribose polymerase PARP (PARP) (1:500; Cell Signaling Technology Inc) and anti‐GAPDH (1:4000) overnight at 4°C. Horseradish peroxidase–conjugated secondary antibodies were diluted 1:5000 and incubated for 1 hour. Protein bands were detected using a chemiluminescence solution, electrochemiluminescence (ECL) kit (Millipore, Massachusetts, MA, USA). Each experiment was repeated three times, and all protein expression was normalized to that of GAPDH.

### 2.15 Cochlear hair cell counts

For quantitative assessment of hair cells, the intact myosin 7a‐positive hair cells were separately counted along the apical, middle and basal turns in each cochlea. The average number of hair cells per 200 µm at different regions in each cochlear explant was calculated from experimental groups. Each experiment was repeated three times, and exact numbers of cochlear explants (n) are indicated in the legends.

### 2.16 Statistical analyses

Statistical analyses were performed using the GraphPad Prism statistical software (version 6; GraphPad Software, Inc, San Diego, CA). Data were shown as mean ± SEM and analysed by one‐way ANOVA. *P* < 0.05 was considered statistically significant.

## 3 RESULTS

### 3.1 Inhibition of ferroptosis protected against cisplatin‐induced loss of cell viability

**FIGURE 1:**
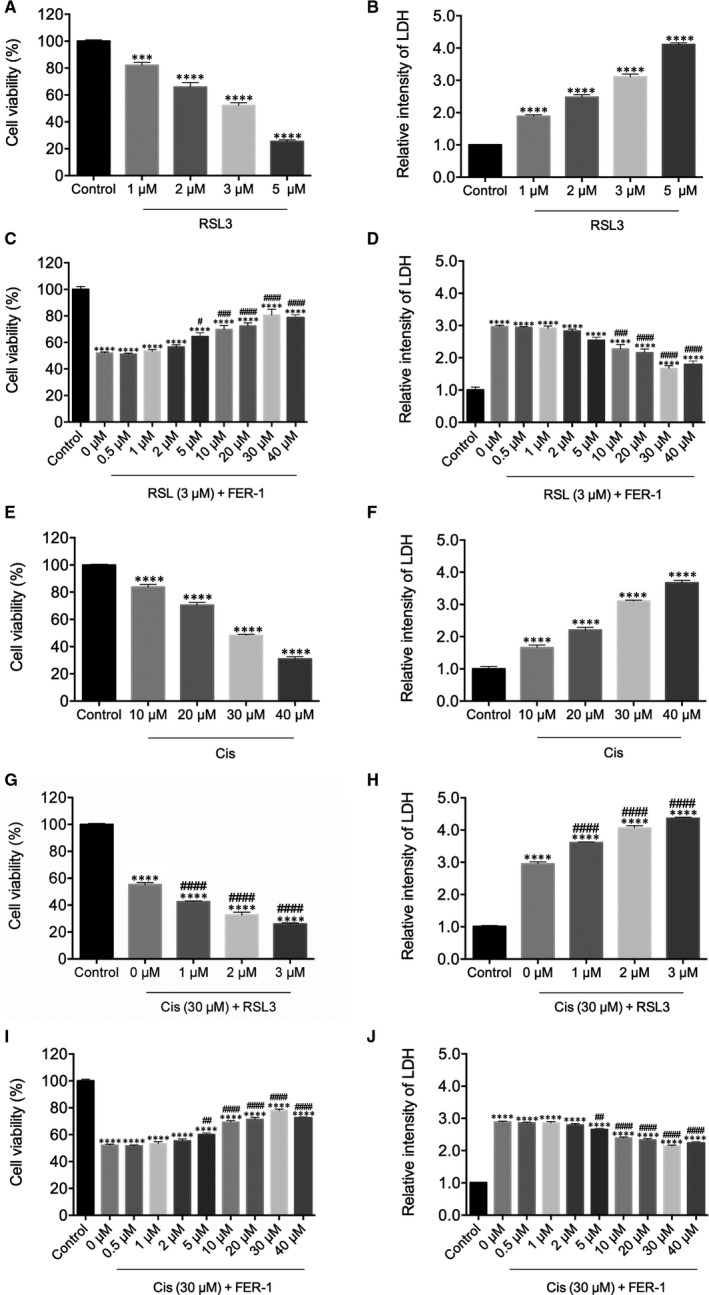
Effects of ferrostatin‐1 (FER‐1) and RSL3 on cell viability in cisplatin‐damaged House Ear Institute‐Organ of Corti 1 cells. A and B, Cells treated with varying concentrations of RSL3 for 24 h were analysed by Cell Counting Kit‐8 (CCK‐8) and lactase dehydrogenase (LDH) assays. (C‐D) Cells were pre‐treated with varying concentrations of FER‐1 for 2 h, followed by addition of 3 μM RSL3 for 24 h and analysed by CCK‐8 and LDH assays. E and F, Cells treated with varying concentrations of cisplatin for 24 h were analysed by CCK‐8 and LDH assays. G and H, Cells were pre‐treated with varying concentrations of RSL3 for 2 h, followed by addition of 30 μmol/L cisplatin for 24 h and analysed by CCK‐8 and LDH assays. I and J, Cells were pre‐treated with varying concentrations of FER‐1 for 2 h, followed by addition of 30 μmol/L cisplatin for 24 h, and analysed by CCK‐8 and LDH assays. All the data represent the mean ± SEM. of three independent experiments. ***P < 0.001, ****P < 0.0001 vs the control group; ^#^ *P* < 0.05, ^##^ *P* < 0.01, ^###^ *P* < 0.001, ^####^ *P* < 0.0001 vs the group treated with RSL3 (C, D) or cisplatin (G‐J) alone

To assess whether RSL3 exposure could induce ferroptosis in hair cells, HEI‐OC1 cells, a widely used auditory hair cell line derived from murine organ of Corti,^20^, ^21^, ^22^, ^23^, ^24^ were exposed to RSL3 at different concentrations for analysis of cell viability and LDH release by CCK‐8 and LDH assays. As seen in Figure 1A,B, exposure to 1, 2, 3 and 5 μmol/L of RSL3 for 24 hours resulted in a dose‐dependent manner of cell viability and LDH release, confirming the ototoxic effect of RSL3 on HEI‐OC1 cells. Based on the cell viability data, we selected 3 μmol/L of RSL3 as an appropriate concentration for the subsequent experiments, as the viability was significantly decreased to ~50% in comparison with the non‐treated control group. Next, to determine if inhibition of ferroptosis could protect HEI‐OC1 cells from RSL3‐induced damage, HEI‐OC1 cells were pre‐treated with increasing concentrations of FER‐1 for 2 hours, and no drugs were added to the control group. The cells were then co‐treated with 3 μmol/L RSL3 for another 24 hours and determined their viability and LDH release. We found an obvious protective effect of FER‐1 above doses of 30 μmol/L, compared with culture treated with RSL3 alone (Figure 1C,D). To further verify that RSL3‐induced HEI‐OC1 cell damage was due to ferroptosis, we checked the effects of other ferroptosis inhibitors on RSL3‐treated cells. As expected, pre‐treatment with DFO, an iron‐chelating agent, or liproxstatin‐1 (Lip‐1), a lipid ROS scavenger, also protected viability of HEI‐OC1 cells upon RSL3 toxicity (Figure S1A,B).

In order to establish the model of cisplatin‐induced injury in HEI‐OC1 cells, increasing concentrations of cisplatin were used to evaluate the cytotoxicity to select the optimum concentration. As shown in Figure 1E, cisplatin treatment for 24 hours was capable of decreasing HEI‐OC1 cell viability in a dose‐dependent manner by CCK‐8 assay in comparison with the non‐treated control group. As the concentration of cisplatin increased, there was a corresponding gradual increase in the release of LDH (Figure 1F). The CCK‐8 assay and LDH release assay showed that cisplatin at a concentration greater than 30 μmol/L markedly induced cytotoxicity; thus, the concentration of 30 μmol/L was chosen as the optimum concentration for HEI‐OC1 cells injury in subsequent experiments. In order to validate the significance of ferroptosis in cisplatin‐induced cell injury, HEI‐OC1 cells were pre‐treated for 2 hours with ferroptosis inducer RSL3 at different concentrations followed by co‐treatment with 30 μmol/L cisplatin for 24 hours. CCK‐8 and LDH assays showed that RSL3 induced a pronounced reduction of cell viability after 24 hours of 30 μmol/L cisplatin exposure in HEI‐OC1 cells in a dose‐dependent manner (Figure 1G,H). In order to test if inhibition of ferroptosis was able to prevent the cisplatin‐induced cytotoxicity, the cells were pre‐treated with FER‐1 for 2 hours and co‐treated with 30 μmol/L cisplatin for another 24 hours. Both CCK‐8 and LDH assays revealed that FER‐1 attenuated the cisplatin‐mediated damage in a concentration‐dependent manner (Figure 1I,J), and FER‐1 at a concentration of 30 μmol/L showed the greatest protective effect against cisplatin‐induced injury; therefore, this concentration chosen as the ideal concentration in subsequent experiments. Furthermore, the decreased cell viability induced by cisplatin was also markedly blocked by pre‐treatment with DFO or Lip‐1 (Figure S1C,D). As previous studies indicate that cisplatin induces apoptosis, we treated HEI‐OC1 cells with different concentrations of a general caspase inhibitor Z‑VAD‑FMK for 2 hours and then with 30 μmol/L cisplatin together with Z‑VAD‑FMK for another 24 hours. The CCK‐8 results showed that Z‑VAD‑FMK at a concentration of 40 μmol/L showed the greatest protective effect against cisplatin‐induced injury, but the number of surviving HEI‐OC‐1 cells was still lower than FER‐1 (30 μmol/L) (Figure S1E). Taken together, these results suggested that ferroptosis played an important role in cisplatin‐induced cell toxicity in HEI‐OC1 cells.

### 3.2 Inhibition of ferroptosis prevented cisplatin‐induced iron overload in HEI‐OC1 cells

**FIGURE 2:**
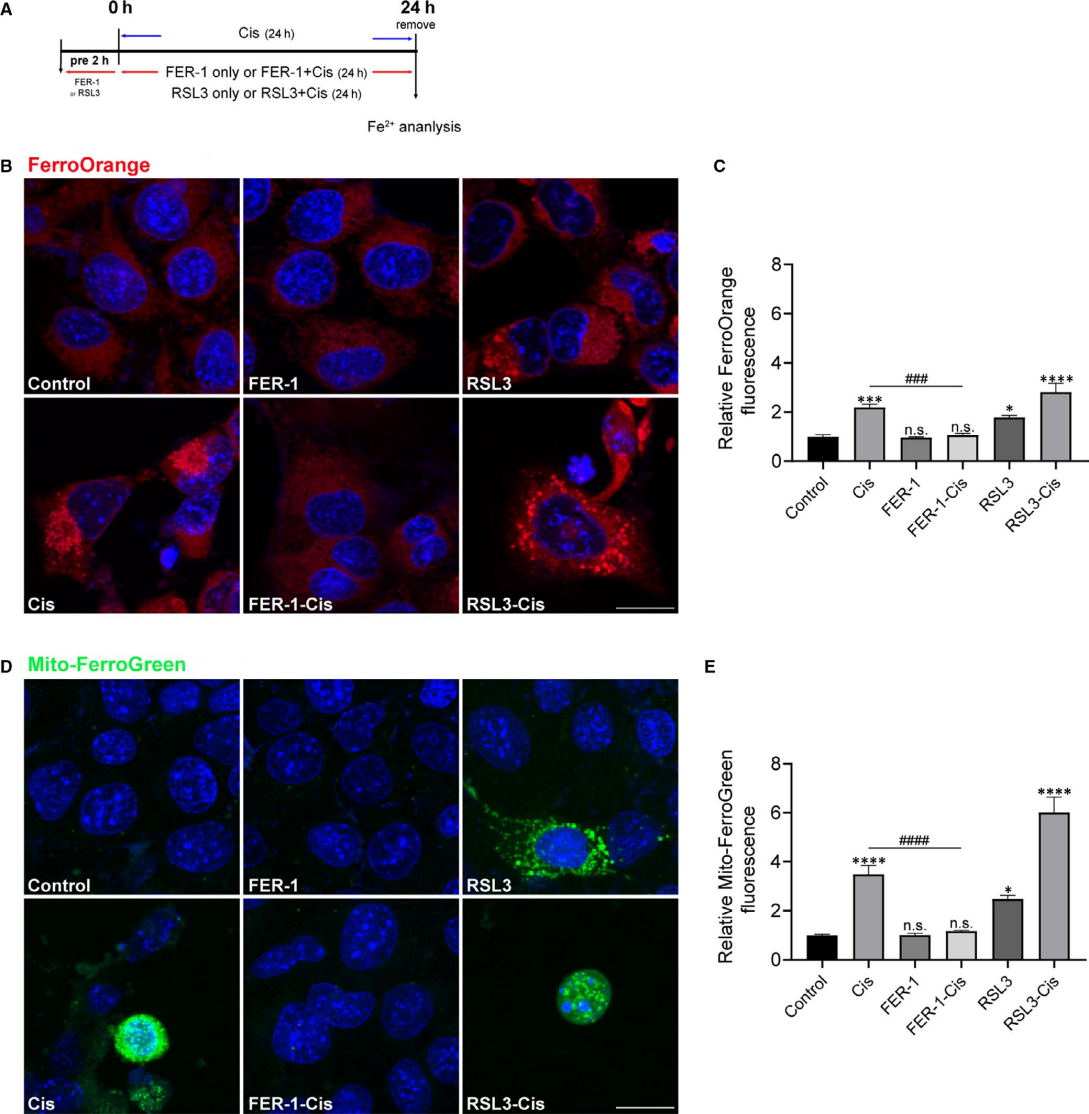
Effect of ferrostatin‐1 (FER‐1) on iron production in cisplatin‐damaged House Ear Institute‐Organ of Corti 1 (HEI‐OC1) cells. A, The experimental workflow. The HEI‐OC1 cells were pre‐treated with 30 μmol/L FER‐1 or 3 μmol/L RSL3 for 2 h and then treated with or without 30 μmol/L cisplatin for another 24 h, or treated with 30 μmol/L cisplatin alone for 24 h. B, Intracellular Fe^2+^ detected by FerroOrange. Scale bar, 20 µm. C, Relative fluorescence intensity of FerroOrange. D, Mitochondrial Fe^2+^ assayed by Mito‐FerroGreen. Scale bar, 20 µm. E, Relative fluorescence intensity of Mito‐FerroGreen. The fluorescence intensity was quantified by ImageJ software. The data are shown as mean ± SEM. *P < 0.05, ***P < 0.001, ****P < 0.0001 and n.s. no significant vs the control group; ^###^ *P* < 0.001 and ^####^ *P* < 0.0001 vs the cisplatin group, n = 8 randomly picked regions from three independent experiments

Given that ferroptosis is mainly dependent on intracellular iron accumulation and lipid peroxidation, we first measured the level of intracellular Fe^2+^ and mitochondrial Fe^2+^ by using FerroOrange and Mito‐FerroGreen probes. The HEI‐OC1 cells were pre‐treated with 30 μmol/L FER‐1 or 3 μmol/L RSL3 for 2 hours and then treated with or without 30 μmol/L cisplatin for another 24 hours, or treated with 30 μmol/L cisplatin alone for 24 hours (Figure 2A). As shown in Figure 2, cisplatin treatment induced excessive iron content in HEI‐OC1 cells as indicated by higher FerroOrange and Mito‐FerroGreen signals than that of normal control cells, while these changes could be remarkably ameliorated by FER‐1 or enhanced by RSL3 pre‐treatment, which suggested that excessive iron content was implicated in the cisplatin‐induced damage in HEI‐OC1 cells. To address the role of ferroptosis in mitochondrial alterations secondary to cisplatin exposure, we used Mito‐FerroGreen to visualize the mitochondrial Fe^2+^, PI probe to label the dead cells, and MitoTracker Red CMXRos probe to label mitochochondria. Confocal microscopy results showed that compared with the undamaged control group, the number of dead cells in the cisplatin group increased significantly (Figure S2), and its mitochondria broke severely, debris increased, co‐localizing with the Mito‐FerroGreen signal (Figure S3), thus suggesting a mitochondria‐based activity of cisplatin‐induced ferroptosis. However, these changes could be remarkably ameliorated by FER‐1 pre‐treatment or enhanced by RSL3 challenge (Figures S2 and S3). Collectively, these results supported a disruptive role of cisplatin‐induced ferroptosis in mitochondrial activity.

### 3.3 Inhibition of ferroptosis ameliorated cisplatin‐induced ROS accumulation in HEI‐OC1 cells

**FIGURE 3:**
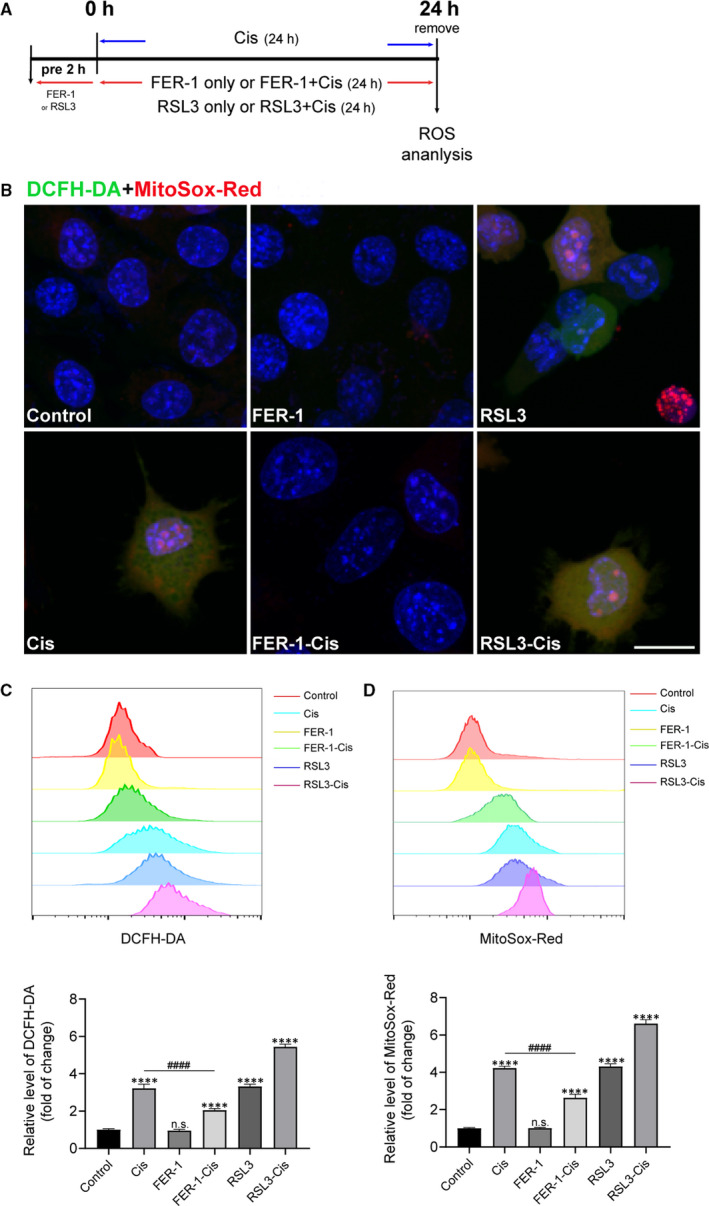
Effect of ferrostatin‐1 (FER‐1) on reactive oxygen species (ROS) generation in cisplatin‐damaged House Ear Institute‐Organ of Corti 1 (HEI‐OC1) cells. A, The experimental workflow. The HEI‐OC1 cells were pre‐treated with 30 μmol/L FER‐1 or 3 μmol/L RSL3 for 2 h and then treated with or without 30 μmol/L cisplatin for another 24 h, or treated with 30 μmol/L cisplatin alone for 24 h. B, Representative images of HEI‐OC1 cells stained by DCFH‐DA and MitoSox‐Red. Scale bar, 20 µm. C and D, Measurement and quantification of intracellular and mitochondrial ROS levels by flow cytometry using DCFH‐DA and MitoSox‐Red fluorescent probes. The data are shown as mean ± SEM. of three independent experiments. ****P < 0.0001 and n.s. no significant vs the control group; ^####^ *P* < 0.0001 vs the cisplatin group

As ROS accumulation is closely related to ferroptosis,^10^, ^25^ we thus determined the production of total ROS by using DCFH‐DA reagent. The staining and flow cytometric analysis results indicated that after cisplatin treatment, the ROS level was significantly increased in HEI‐OC1 cells pre‐treated with RSL3 compared with the cisplatin only group. In contrast, pre‐treatment of the cells with FER‐1 considerably ameliorated the cisplatin‐mediated overproduction of ROS (Figure 3). The levels of mitochondrial ROS were evaluated by MitoSox‐Red staining, in agreement with changes in intracellular ROS levels, FER‐1 pre‐treatment could abolish the mitochondrial ROS level that was induced by cisplatin (Figure 3). Altogether, these results indicated that ROS was indispensable for cisplatin‐induced ferroptosis.

**FIGURE 4:**
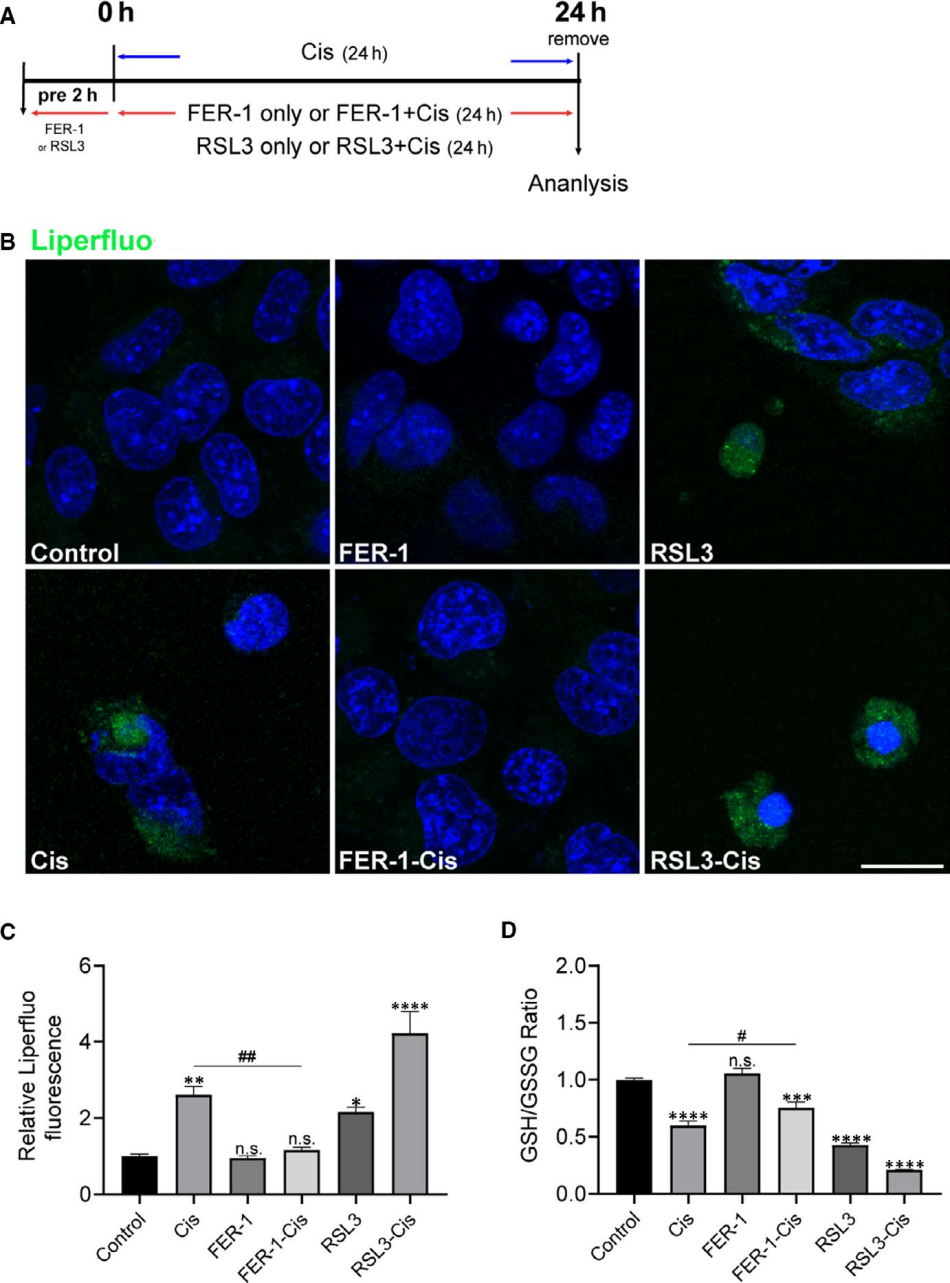
Effects of ferrostatin‐1 (FER‐1) on lipid reactive oxygen species (ROS) production and ratio of glutathione (GSH) and glutathione disulphide (GSSG) in cisplatin‐damaged House Ear Institute‐Organ of Corti 1 (HEI‐OC1) cells. A, The experimental workflow. The HEI‐OC1 cells were pre‐treated with 3 μmol/L RSL3 or 30 μmol/L FER‐1 for 2 h and then treated with or without 30 μmol/L cisplatin for another 24 h, or treated with 30 μmol/L cisplatin alone for 24 h, and then lipid ROS was detected by Liperfluo. B, Representative images of Liperfluo staining. Scale bar, 20 µm. C, The fluorescence intensity was quantified by ImageJ software. The data are shown as mean ± SEM. of three independent experiments. *P < 0.05, **P < 0.01, ****P < 0.0001 and n.s. no significant vs the control group; ^##^ *P* < 0.01 vs the cisplatin group. D, GSH/GSSG assay. The data is shown as mean ± SEM. of three independent experiments. ***P < 0.001, ****P < 0.0001 and n.s. no significant vs the control group; ^#^ *P* < 0.05 vs the cisplatin group

Using Liperfluo staining, we next detected lipid peroxidation in HEI‐OC1 cells. From Figure 4, Liperfluo signal significantly increased in cisplatin‐treated cells, indicating that cisplatin was able to induce lipid peroxidation in HEI‐OC1 cells. Meanwhile, a reduction in lipid peroxidation was observed in cells pre‐treated with FER‐1, compared with cisplatin alone (Figure 4B,C). Additionally, when we used another C11‐BODIPY^581/591^ probe as a lipid peroxide indicator,^10^ FER‐1 pre‐treatment significantly decreased the lipid peroxide level that was increased by cisplatin (Figure S4). Treatment with FER‐1 only was unable to produce an increase of both Liperfluo and C11‐BODIPY^581/591^ markers. Given that GSH plays key roles in exerting cellular antioxidant functions, we next investigated the mechanism underlying the otoprotective effect of FER‐1 by testing GSH/GSSG ratio. As shown in Figure 4D, after treatment with cisplatin, the GSH/GSSG ratio in HEI‐OC1 cells markedly decreased in contrast to the control group. However, pre‐treatment with FER‐1 significantly increased the GSH/GSSG ratio compared with the cisplatin group (Figure 4D). Taken together, these results hinted that cisplatin disrupted cellular antioxidant capacity and induced ferroptosis in HEI‐OC1 cells.

### 3.4 Inhibition of ferroptosis prevented cisplatin‐induced breakdown of MMP in HEI‐OC1 cells

**FIGURE 5:**
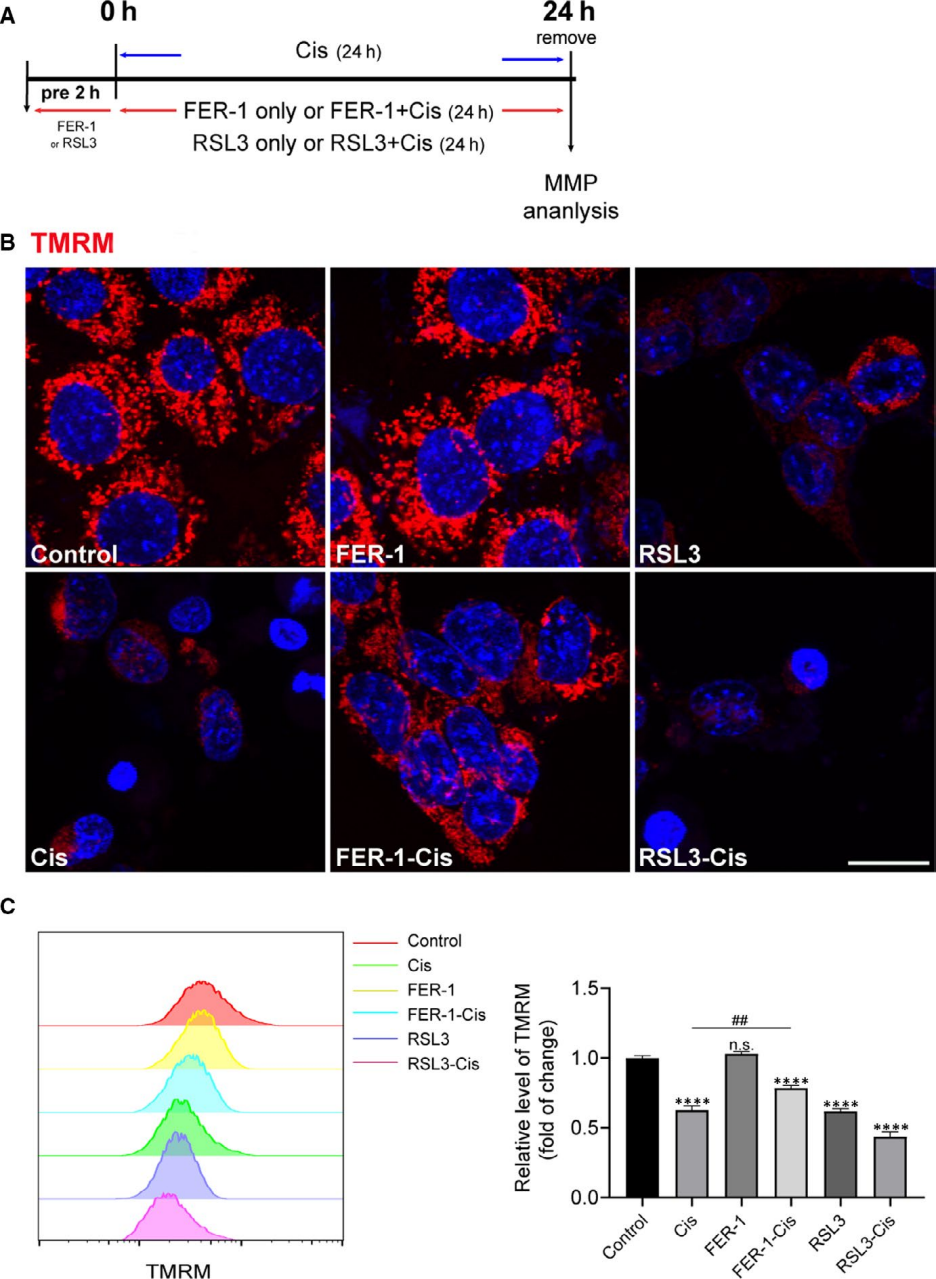
Effect of ferrostatin‐1 (FER‐1) on mitochondrial membrane potential (MMP) in cisplatin‐damaged House Ear Institute‐Organ of Corti 1 (HEI‐OC1) cells. A, The experimental workflow. The HEI‐OC1 cells were pre‐treated with 3 μmol/L RSL3 or 30 μmol/L FER‐1 for 2 h and then treated with or without 30 μmol/L cisplatin for another 24 h, or treated with 30 μmol/L cisplatin alone for 24 h, and then MMP was detected by TMRM. B, Representative images of HEI‐OC1 cells stained with TMRM. Scale bar, 20 µm. C, Measurement and quantification of MMP by flow cytometry. The data are shown as mean ± SEM. of three independent experiments. ****P < 0.0001 and n.s. no significant vs the control group; ^##^ *P* < 0.01 vs the cisplatin group

To investigate the effect of ferroptosis on MMP in cisplatin‐damaged HEI‐OC1 cells, MMP was measured by TMRM staining. As shown in Figure 5, the TMRM fluorescence reduced significantly in the cisplatin group, compared with the control group, indicating that cisplatin challenge provoked a loss of membrane potential; and exacerbated vanishing membrane potential was observed after cisplatin and RSL3 treatment (Figure 5). Conversely, FER‐1 pre‐treatment protected the cells against mitochondrial damage after exposure of cisplatin, as evidenced by enhanced fluorescence intensity (Figure 5).

### 3.5 Inhibition of ferroptosis protected cochlear hair cells against cisplatin‐induced damage

**FIGURE 6:**
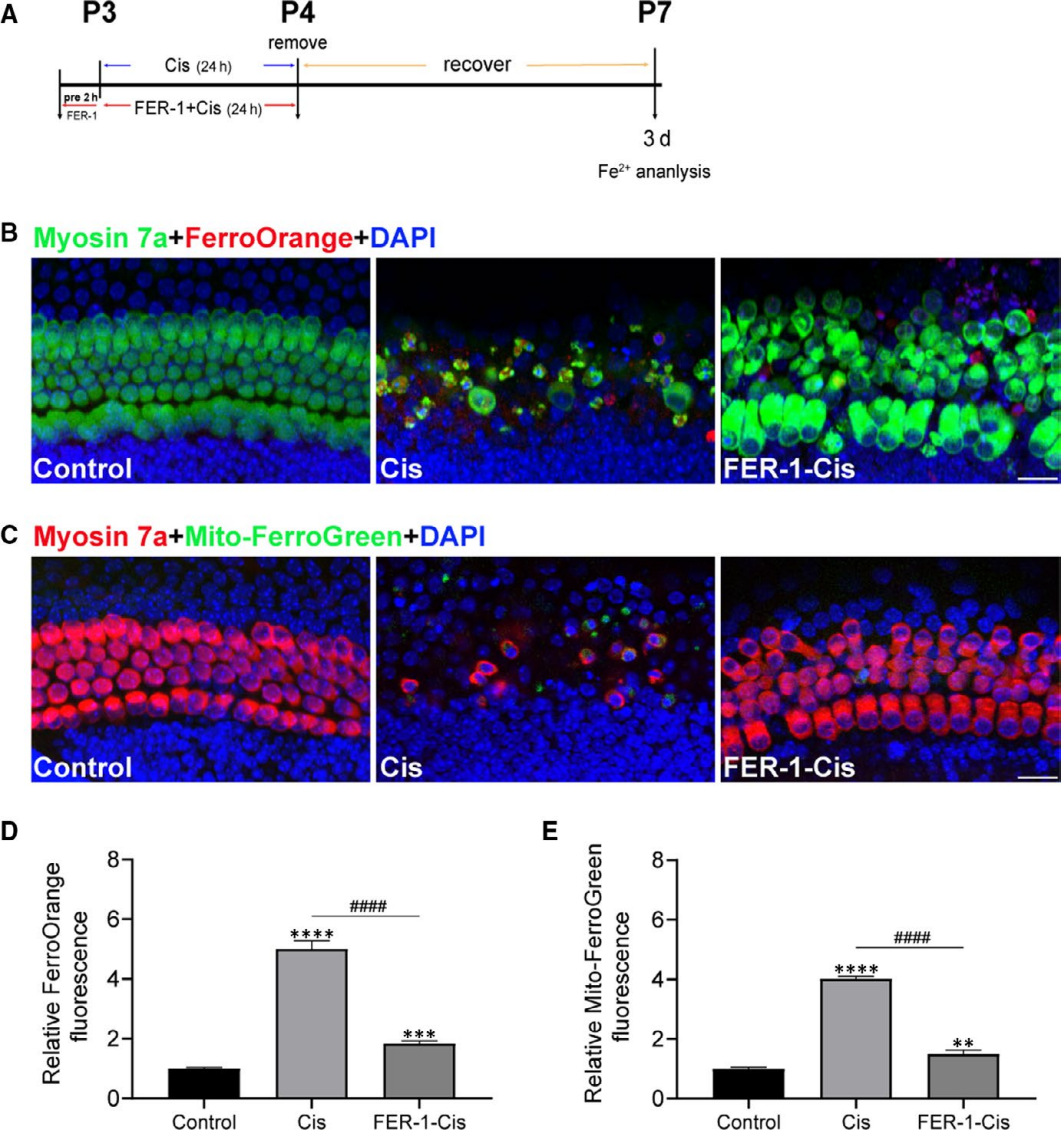
Effect of ferrostatin‐1 (FER‐1) on iron accumulation in cisplatin‐damaged cochlear hair cells. A, The experimental workflow. Cochlear explants were treated with 30 μmol/L cisplatin alone (Cis) for 24 h, or pre‐treatment with 30 μmol/L FER‐1 for 2 h and addition of 30 μmol/L cisplatin for 24 h followed by 3 d recovery. B, Representative images of myosin 7a (green) and FerroOrange (red) staining of middle cochlear turns from different groups. Scale bar, 20 µm. C, Representative images of myosin 7a (red) and Mito‐FerroGreen (green) staining of middle cochlear turns from different groups. Scale bar, 20 µm. D and E, Relative fluorescence intensity of FerroOrange and Mito‐FerroGreen. The data are shown as mean ± SEM. **P < 0.01, ***P < 0.001, ****P < 0.0001 vs the control group; ^####^ *P* < 0.0001 vs the cisplatin group, n = 12‐18 cochlear explants from three independent experiments

Moreover, we strengthened the findings from HEI‐OC1 cells in organotypically cultured cochlear explants from P2 C57BL/6 mice in vitro.^26^ Cochlear explants were treated with culture media, 30 μmol/L cisplatin alone, or pre‐treatment with 30 μmol/L FER‐1 for 2 hours and addition of 30 μmol/L cisplatin for another 24 hours. After FER‐1 and cisplatin were removed, the explants were recovered for various durations (0, 1, 2 and 3 days), and the levels of intracellular and mitochondrial Fe^2+^ were examined by FerroOrange and Mito‐FerroGreen probes. As expected, experimental results demonstrated that cisplatin resulted in a time‐dependent increase in intracellular and mitochondrial Fe^2+^ in cultured cochlea explants (Figures S5 and S6). The maximal induction of both signals was found at 3 days after cisplatin exposure, and this increase was significantly inhibited by FER‐1 pre‐treatment (Figure 6). Thus, 3 days recovery after cisplatin exposure was used to establish the cisplatin model in subsequent experiments. In order to deeply investigate the mechanism of ferroptosis in cisplatin‐induced damage, we examined the protein markers for ferroptosis (GPX4) and apoptosis (cleaved‐PARP). Western blotting data showed that cisplatin not only induced cleaved‐PARP expression, but inhibited the protein level of GPX4, while FER‐1 pre‐treatment reversed GPX4 expression in whole‐cell extracts from cochlear explants, but not cleaved‐PARP (Figure S6A). To further investigate whether the otoprotective effects of FER‐1 contributed to cell apoptosis secondary to cisplatin exposure, we analysed apoptosis in cultured cochleae by cleaved caspase‐3 staining, and hair cells were labelled with another specific hair cell marker, parvalbumin.^27^, ^28^ Immunofluorescence results showed that cisplatin significantly increased the number of cleaved caspase‐3 positive cells compared with the undamaged control group, and this effect was not reversed by the pre‐treatment with FER‐1 (Figure S6B,C).

**FIGURE 7:**
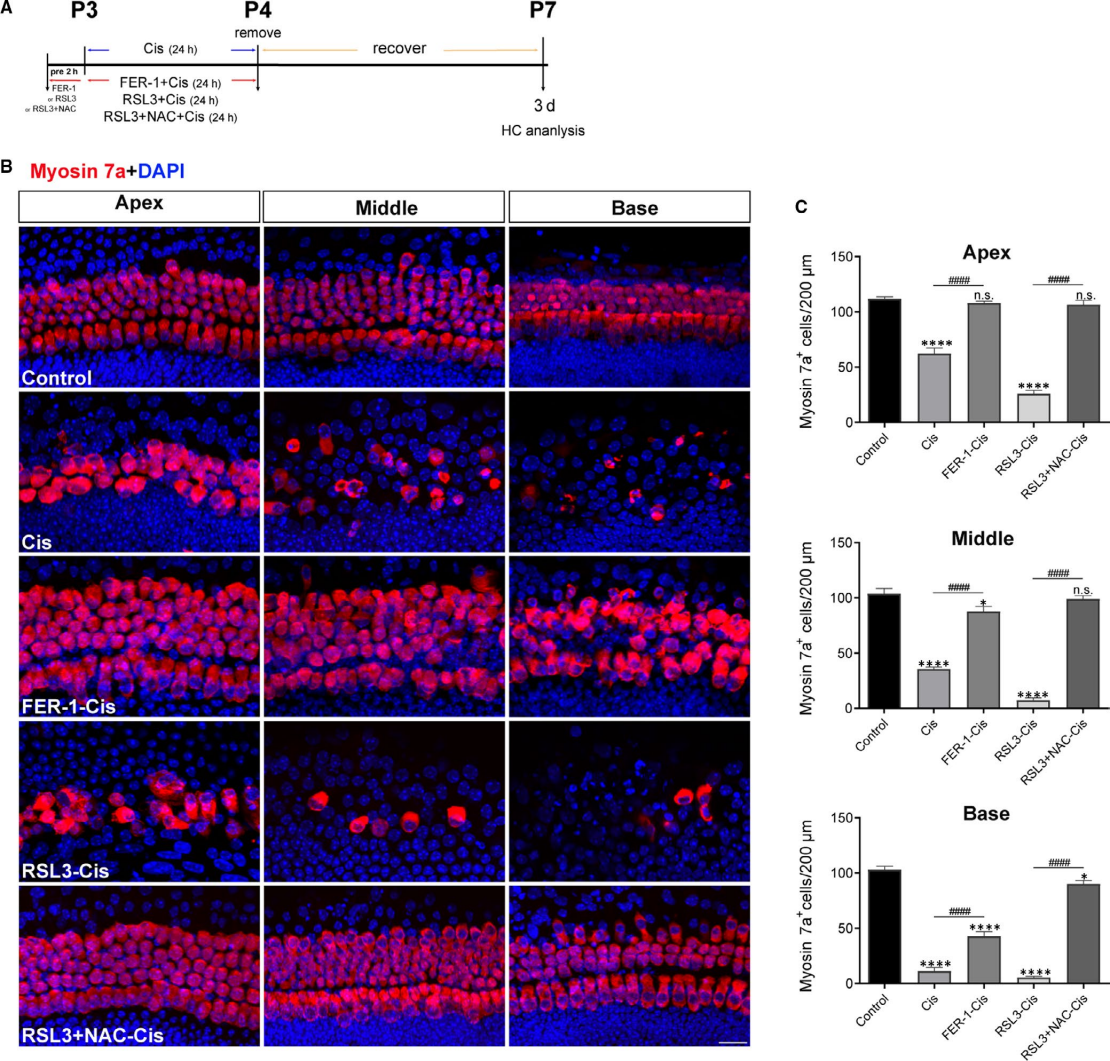
Effect of ferrostatin‐1 (FER‐1) on cisplatin‐induced cochlear hair cell damage. A, The experimental workflow. Cochlear explants were treated with 30 μmol/L cisplatin alone for 24 h, or pre‐treated with 30 μmol/L FER‐1, 3 μmol/L RSL3 or 3 μmol/L RSL3 + 5 mmol/L NAC for 2 h and addition of 30 μmol/L cisplatin for 24 h followed by 3 d recovery. B, Representative images of cochlear hair cells stained by myosin 7a (red). Scale bar, 20 µm. C, Quantification of myosin 7a‐positive hair cells in the apical, middle, and basal turns of different groups. The data are shown as mean ± SEM. *P < 0.05, ****P < 0.0001 and n.s. no significant vs the control group; ^####^ *P* < 0.0001, n = 12 cochlear explants from three independent experiments

**FIGURE 8:**
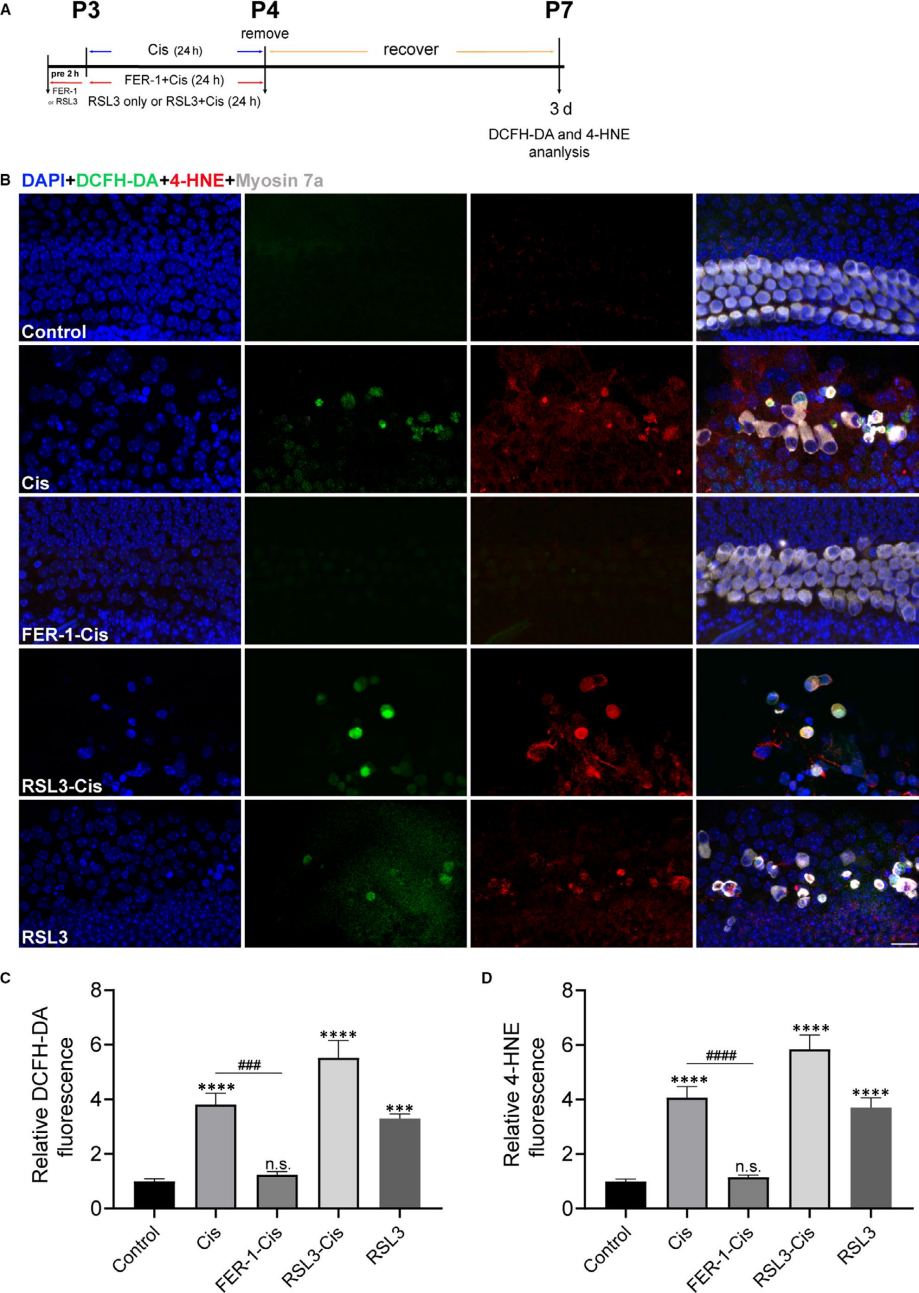
Effects of ferrostatin‐1 (FER‐1) on reactive oxygen species level and 4‐HNE in cisplatin‐damaged cochlear hair cells. A, The experimental workflow. Cochlear explants were treated with only 30 μmol/L cisplatin (24 h), 3 μmol/L RSL3 (26 h), or pre‐treated with 30 μmol/L FER‐1 or 3 μmol/L RSL3 for 2 h and addition of 30 μmol/L cisplatin for 24 h followed by 3 d recovery. B, Representative images of cochlear (middle turn) hair cells stained by DCFH‐DA (green), 4‐HNE (red) and myosin 7a (grey). Scale bar, 20 µm. C and D, Relative fluorescence intensity of DCFH‐DA and 4‐HNE. The data are shown as mean ± SEM. ***P < 0.001, ****P < 0.0001 and n.s. no significant vs the control group; ^###^ *P* < 0.001, ^####^ *P* < 0.0001 vs the cisplatin group, n = 6 cochlear explants from three independent experiments

To identify whether FER‐1 pre‐treatment also protected hair cells in the cochlear explants against cisplatin‐induced damage. Hair cells with normal nuclei and labelled with myosin 7a, a hair cell marker,^29^, ^30^ were counted. Immunofluorescence staining showed that cisplatin administration significantly decreased the number of sensory hair cells in cochlear explants (Figure 7). In contrast, FER‐1 pre‐treatment significantly increased the survival of hair cells in cisplatin‐damaged organs of Corti (Figure 7). Given the importance of ROS generation in cisplatin‐induced ferroptosis, ROS levels in cochlear explants were monitored upon different treatment using DCFH‐DA probe. The result showed that the explants exposed to cisplatin exhibited considerable increased DCFH‐DA fluorescent intensity compared with the undamaged control group. In contrast, FER‐1 pre‐treatment significantly suppressed the cisplatin‐induced ROS generation compared with the cisplatin alone (Figure 8). Of note, increased ROS production was also observed in cochlear hair cells treated with RSL3 alone, similar to the cisplatin group, and markedly enhanced by cisplatin exposure (Figure 8). We also examined the lipid peroxidation product 4‐HNE by immunostaining, an effective biomarker for oxidative damage.^31^ As results, cisplatin treatment increased the expression of 4‐HNE in hair cells, and RSL3 notably enhanced the cisplatin‐induced increase level of 4‐HNE; however, FER‐1 pre‐treatment significantly reduced the level of 4‐HNE in hair cells as compared to the explants only treated with cisplatin alone, indicating that ferroptosis inhibition indeed reduced the oxidative damage to cochlear hair cells (Figure 8). To further definitively determine whether ROS generation contributed to the increased sensitivity of cochlear hair cells to cisplatin‐induced damage, cochlear explants were pre‐treated with 3 μmol/L RSL3 alone or 3 μmol/L RSL3 with 5 mmol/L ROS scavenger NAC^32^ for 2 hours and addition of 30 μmol/L cisplatin for another 24 hours followed by 3 days recovery. The results indicated that NAC considerably ameliorated the RSL3‐induced significant loss of hair cells in cisplatin‐damaged explants (Figure 7), indicating that ROS generation was indispensable for the process of cisplatin‐induced ferroptosis.

## 4 DISCUSSION

As the roles of ferroptosis in the pathological situations have been recently investigated, the importance of ferroptosis in ototoxic pharmaceutical agents‐mediated hearing loss is increasing. We first demonstrated that ferroptosis plays a pivotal role in the development and progression of cisplatin‐induced hair cell injury, and inhibition of ferroptosis potentially protected hair cells from cisplatin‐mediated damage through mechanisms involving preservation of mitochondrial parameters, such as maintaining MMP and inhibiting ROS production.

Although the production of excessive ROS is considered to be the major cause of cisplatin‐induced ototoxicity; increasing ROS production leads to downstream cytokine deprivation, mitochondrial dysfunction and eventually leading to cell death,^33^, ^34^, ^35^, ^36^ the exact molecular mechanism of cisplatin ototoxicity is still not fully understood. Ferroptosis is a type of newly discovered programmed cell death dependent on iron^37^; it is distinct from other cell death forms such as apoptosis, autophagy or necrosis.^10^ Ferroptosis has been implicated in multiple pathological situations, such as cancers^38^ and neurodegenerative diseases.^17^ Recent studies have indicated that triggering ferroptosis in cancer cells is a promising approach to increase the sensitivity to cisplatin.^39^, ^40^, ^41^ For example, under the stress stimuli, the promotion of ferroptosis by ferroptosis inducer reactivated ferroptosis in osteosarcoma cells and consequently enhanced the sensitivity to cisplatin.^39^ According to the earlier observation of Song and Schacht et al^42^ iron availability is a critical factor in aminoglycoside‐induced ototoxicity and iron chelators are more efficient than numbers of radical scavengers in attenuating aminoglycoside‐induced hearing loss. Studies from these mechanisms provided promising therapeutic prevention for the amelioration of ototoxic drugs‐induced ototoxicity by use of iron chelators. However, the role of ferroptosis in the prevention of cisplatin‐induced ototoxicity remains unsolved.

In this study, we first analysed the correlation between cisplatin and ferroptosis in HEI‐OC1 cells. Both CCK‐8 and LDH analysis showed that cisplatin significantly reduced cell viability compared the normal control cells. In contrast, FER‐1 treatment prevented the cisplatin‐mediated cytotoxicity in a concentration‐dependent manner. Of note, the toxicity induced in HEI‐OC1 cells by cisplatin could also be rescued by apoptosis inhibitor, but the cell viability was still lower than FER‐1 pre‐treatment. This finding supports the conclusion that ferroptosis plays an important role in cisplatin‐induced cell toxicity in HEI‐OC1 cells. Generally, ferroptosis accompanied with intracellular accumulation of excessive iron and lipid peroxidase.^43^ Initially, we proved that iron overload could be induced by cisplatin exposure. We employed two assays detecting Fe^2+^: the FerroOrange staining reflects intracellular Fe^2+^ and the Mito‐FerroGreen staining assesses mitochondrial Fe^2+^, respectively. Our data showed that cisplatin induced a significant increase in both intracellular and mitochondrial ferrous iron level in HEI‐OC1 cells, which could be remarkably reversed by ferroptosis inhibitor FER‐1 treatment or enhanced by ferroptosis inducer RSL3 treatment. In addition to iron overburden, lipid peroxidation is another fundamental element in ferroptosis, we next explored whether the cisplatin‐treated HEI‐OC1 cells were accompanied by lipid peroxide accumulation. Different assays have been recently developed to target the multiple intermediate products generated during the lipid peroxidation (LPO) synthetic biosynthetic pathway (ie Liperfluo, C11‐BODIPY^581/591^). Liperfluo, a perylene derivative containing oligooxyethylene, directly reacts with (phospho) lipid hydroperoxides to form fluorescent product Liperfluo‐OX; it responds to membrane lipid hydroperoxides and reports intracellular sites of lipid hydroperoxide accumulation.^44^, ^45^ Moreover, due to its high solubility in various organic solvents, such as ethanol and dimethylsulphoxide, this probe is extremely useful for the imaging of lipid hydroperoxides in living cells.^45^ In the current study, we exposed cultured HEI‐OC1 cells to cisplatin and estimated LPO production based on the Liperfluo signal. Liperfluo signal significantly increased in the presence of cisplatin; this illustrated that cisplatin‐induced damage in cultured HEI‐OC1 cells is directly due to iron‑mediated lipid peroxidation. In contrast, the increment of lipid LPO in response to cisplatin injury was remarkably ameliorated by FER‐1 treatment. Additionally, we further validated this finding using another lipid peroxidation‐sensitive dye (C11‐BODIPY^581/591^) confirming the data obtained by Liperfluo. C11‐BODIPY^581/591^ is a fatty acid analogue and binds to hydroxyl and superoxide radicals; it is a fluorescent radio‐probe for indexing lipid peroxidation and antioxidant efficacy in model membrane systems. C11‐BODIPY^581/591^ has a high quantum yield of fluorescence emission, assuring a good signal measurement, and it has a good photo‐stability and its fluorescence emission is virtually insensitive to environmental changes such as solvent polarity or pH. Oxidation of the C11‐BODIPY^581/591^ induces a shift of the fluorescence emission maximum from red (non‐oxidized) to green (oxidized) fluorescence.^46^ Furthermore, C11‐BODIPY^581/591^ was reported to be very sensitive to free radicals formed by the decomposition of hydroperoxides, but not to hydroperoxides themselves.^16^, ^46^, ^47^, ^48^ Use of both the Liperfluo and C11‐BODIPY^581/591^ assays allow for detection of LPO, but in contrast to C11‐BODIPY^581/591^, which does not react with a lipid hydroperoxide, Liperfluo is the only compound that can specifically detect lipid peroxides; thus, the Liperfluo assay may be the most closely related to the mechanisms of ferroptosis. Moreover, previous studies have provided evidence that GSH serves a major role in protecting cells against damage, while GSH depletion could induce an iron‐dependent accumulation of lipid peroxidation, eventually triggering ferroptotic cell death.^16^ Here, we discovered that cisplatin significantly inhibited the GSH/GSSG ratio in HEI‐OC1 cells compared to the undamaged controls; the decrease, however, was reversed by FER‐1 treatment. Together, these findings illustrated that cisplatin led to ferroptosis through enhancing iron overburden and lipid peroxidation, reducing GSH in HEI‐OC1 cells.

Accumulating studies showed that cisplatin ototoxicity was closely related to the enhanced ROS production in the inner ear.^49^, ^50^ Cisplatin triggers overproduction of ROS in the cochlea resulting in sensory epithelial cell death.^33^ To investigate the underlying mechanism of the protective effect of FER‐1 on HEI‐OC1 cells, we examined the intracellular and mitochondrial ROS. Our results showed that cisplatin exposure remarkably increased the levels of intracellular and mitochondrial ROS compared with control cells, and this may be explained by the fact that cisplatin‐mediated iron overload could immediately induce ROS generation. In contrast, FER‐1 treatment significantly inhibited the accumulation of intracellular and mitochondrial ROS caused by cisplatin in HEI‐OC1 cells. These results confirmed the role of ROS in cisplatin‑induced ferroptosis and highlighted the effect of ferroptosis inhibitors in improving cisplatin‑induced hair cell loss.

Mitochondria is a key regulator of cellular processes, and mitochondrion dysfunction plays an important role in cell death. Increasing evidence suggested that the mitochondria was important sources of ROS generation that led to cisplatin‐induced ototoxicity.^51^ In this research, to investigate whether ferroptosis inhibition has a role in the modulation of mitochondrial dysfunction induced by cisplatin, MMP (△Ψm), an important index of mitochondrial function, was examined. We loaded the HEI‐OC1 cells with the △Ψm indicator TMRM and assessed the role of ferroptosis in mitochondrial dysfunction secondary to cisplatin exposure by evaluating △Ψm with and without FER‐1 pre‐treatment. The results indicated that cisplatin treatment resulted in a significant loss of MMP and damaged the structure of mitochondria, while FER‐1 significantly mitigated these effects induced by cisplatin. These results indicated that inhibition of ferroptosis plays a positive role in mitigating hair cell death secondary to cisplatin exposure through preserving mitochondrial function.

Using an organotypically cultured organ of Corti model, we confirmed the protective effect of ferroptosis inhibitor against cisplatin‐induced ototoxicity in neonatal mouse cochlear explants. After cisplatin administration, extensive degeneration of HCs was observed, and this cisplatin‐mediated ototoxicity propagated from the base of the cochlea to the apex, in accordance with previous reports.^52^ Treatment with FER‐1 significantly attenuated cisplatin‐induced loss of hair cells. Of note, our data showed that cochlear explants co‐treatment with cisplatin and RSL3 had notably decreased hair cells compared with cisplatin treatment alone, indicating that RSL3 notably potentiated the cytotoxic effects of cisplatin in cochlear hair cells. The impact of overproduction of ROS induced by cisplatin on ferroptosis was further assessed by treating cisplatin‐damaged cochlear hair cells with RSL3 both without and in combination with radical scavenger NAC for 24 hours, respectively, we found significant protection by application of NAC; these findings were in consonance with those studies showed that NAC protected cochlea against cisplatin ototoxicity.^53^, ^54^

Glutathione peroxidase 4, a GSH‐dependent enzyme, is one of the most important antioxidant enzymes that selectively catalyses lipid hydroperoxides, reducing lipid hydroperoxides to lipid alcohols.^55^, ^56^, ^57^ Previous studies have reported that inhibition of GPX4 activity leads to rapid lipid peroxide accumulation, triggering an iron‐dependent ferroptotic cell death,^38^, ^58^ and deletion of GPX4 in mice is embryonic lethal.^16^ In the present study, our data suggested that cisplatin toxicity was not only a result of increase in cleaved‐PARP protein expression but, was also attributed to a remarkable reduction of protein levels of GPX4 within 24 hours of cisplatin exposure, while addition of FER‐1 reversed GPX4 expression, but had no effect on cleaved‐PARP. These observations, in combination with our observations that iron overburden, ROS/LPO production, mitochondrial dysfunction secondary to cisplatin exposure were largely ameliorated by FER‐1 pre‐treatment, imply an important role for ferroptosis in cisplatin‐induced ototoxicity, and the administration of ferroptosis inhibitor, significantly prevented the ferroptotic cell death secondary to cisplatin exposure.

It is important to note that although inhibition of ferroptosis in vitro has provided highly promising protective effects, further exact mechanism and in vivo mouse model studies are needed. Although we showed a critical function and underlying mechanisms of ferroptosis in cisplatin‐induced ototoxicity in vitro and demonstrated that its inhibition may be a potential therapeutic approach for treating cisplatin‐induced hearing loss, the effects of ferroptosis inhibitor on some key proteins related to cell survival or other mitochondrial parameters after cisplatin is currently unclear, which would require further examination.

In conclusion, the present study reveals that ferroptosis plays a vital role in cisplatin‐induced hair cell damage. Inhibition of ferroptosis antagonizes cisplatin‐induced ototoxicity through inhibiting oxidative toxicity and mitochondrial dysfunction in both cultured auditory HEI‐OC1 cells and neonatal mouse cochlear explants. Our findings suggest that ferroptosis inhibitor might serve as potential otoprotectant for the treatment of cisplatin‐induced hearing loss.

## CONFLICT OF INTERESTS

The authors declare no competing financial interests.

## AUTHOR CONTRIBUTION

**Honglin Mei:** Conceptualization (supporting); Project administration (lead); Supervision (supporting); Validation (equal). **Liping Zhao:** Investigation (lead); Methodology (lead); Project administration (lead); Supervision (supporting). **Wen Li:** Methodology (equal); Project administration (lead); Validation (equal); Visualization (equal). **Zhiwei Zheng:** Project administration (supporting); Validation (equal). **Dongmei Tang:** Funding acquisition (supporting); Project administration (supporting); Validation (equal). **Xiaoling Lu:** Funding acquisition (supporting); Project administration (supporting); Validation (equal). **Yingzi He:** Conceptualization (lead); Funding acquisition (lead); Project administration (equal); Supervision (lead); Writing‐original draft (lead); Writing‐review & editing (lead).

## AUTHOR CONTRIBUTIONS

HM and YH designed the study. HM, WL, LZ, ZZ, DT and XL performed experiments. WL, LZ and YH analysed data. YH wrote the manuscript. HM, WL, LZ, ZZ, DT, XL and YH commented on the manuscript.

## Supporting information

Fig S1‐6

## Data Availability

The data used to support the findings of this study are available from the corresponding author upon request.
