# Supplementary material for: Inhibition of ferroptosis protects House Ear Institute‐Organ of Corti 1 cells and cochlear hair cells from cisplatin‐induced ototoxicity
Source: J Cell Mol Med. 2020 Sep 14;24(20):12065–81. doi: 10.1111/jcmm.15839 (PMC7579698; doi:10.1111/jcmm.15839)
Supplement: Supplementary file 1 — Fig S1‐6 [file JCMM-24-12065-s001.docx]

**Inhibition of ferroptosis protects House Ear Institute‐Organ of Corti 1 cells and cochlear hair cells from cisplatin-induced ototoxicity**

Honglin Mei ^1,2^*, Liping Zhao ^1,2^*, Wen Li ^1,2^*, Zhiwei Zheng ^1,2^, Dongmei Tang ^1,2^, Xiaoling Lu ^1,2^, Yingzi He ^1,2#^

^1^ ENT institute and Department of Otorhinolaryngology, Eye & ENT Hospital, Fudan University, Shanghai, 200031, China

^2^ NHC Key Laboratory of Hearing Medicine (Fudan University), Shanghai, 200031, China

* Honglin Mei, Liping Zhao and Wen Li contributed equally to this work.

# **Correspondence should be addressed to**:

Yingzi He, ENT institute and Otorhinolaryngology Department of Affiliated Eye and ENT Hospital, State Key Laboratory of Medical Neurobiology, Fudan University, 83 Fenyang Road, Shanghai, 200031, China; Tel: +86-21-64377134; E-mail: yingzihe09611@126.com

**Running title:** The role of ferroptosis in cisplatin-mediated ototoxicity

**Supplementary Figures**

**
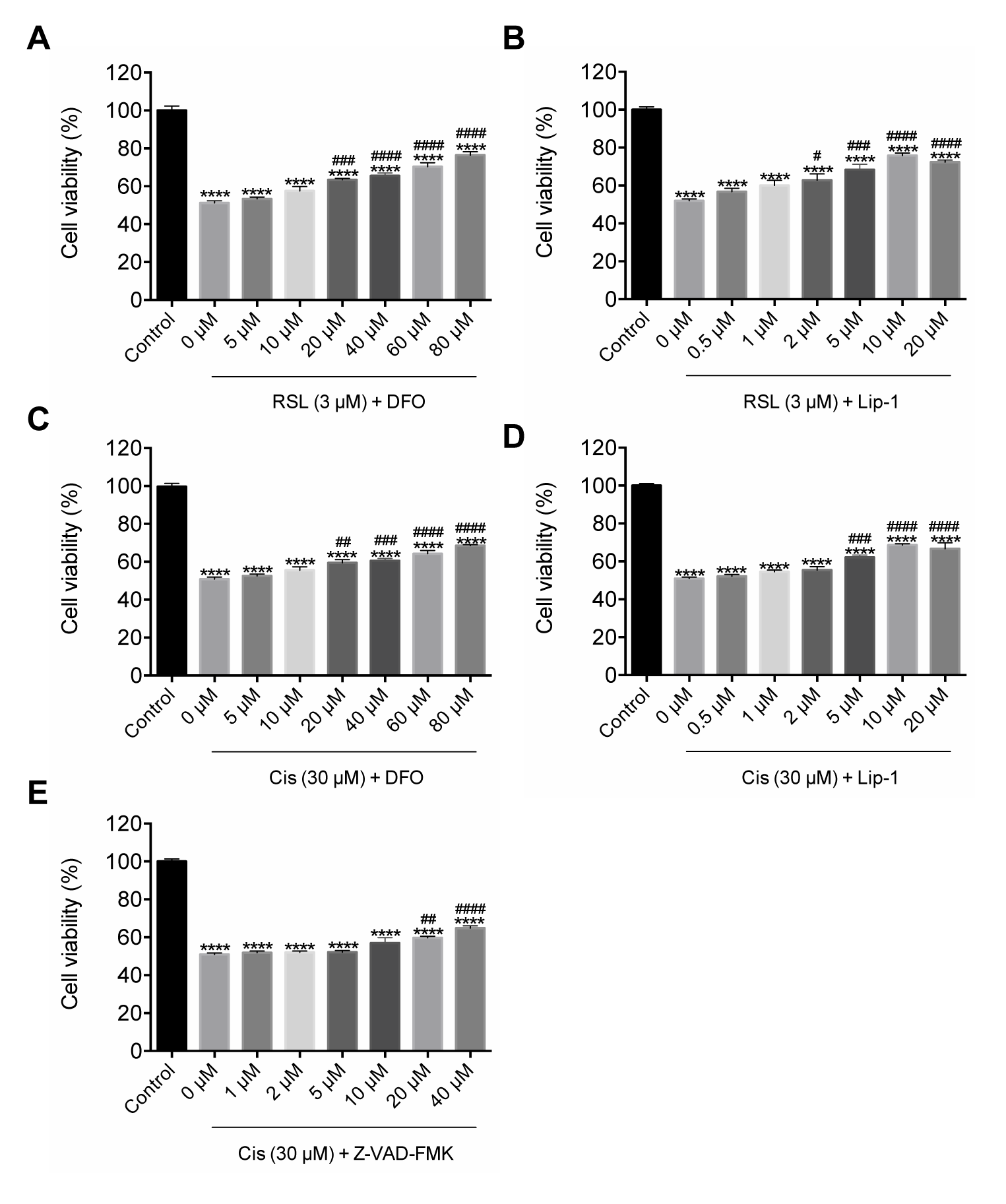
**

**Supplementary Figure 1**

Effects of DFO, Lip-1, or Z‑VAD‑FMK on cell viability in HEI-OC1 cells. (A-B) Cells were pre-treated with varying concentrations of DFO or Lip-1 for 2 h, followed by addition of 3 μM RSL3 for 24 h, and analyzed by CCK-8 assay. (C-E) Cells were pre-treated with varying concentrations of DFO, Lip-1, or Z‑VAD‑FMK for 2 h, followed by addition of 30 μM cisplatin for 24 h, and analyzed by CCK-8 assay. All the data represent the mean ± SEM. of three independent experiments. *****p* < 0.0001 vs. the control group; ^#^*p* < 0.05, ^##^*p* < 0.01, ^###^*p* < 0.001, ^####^*p* < 0.0001 vs. the group treated with RSL3 (A and B) or cisplatin (C-E) alone.


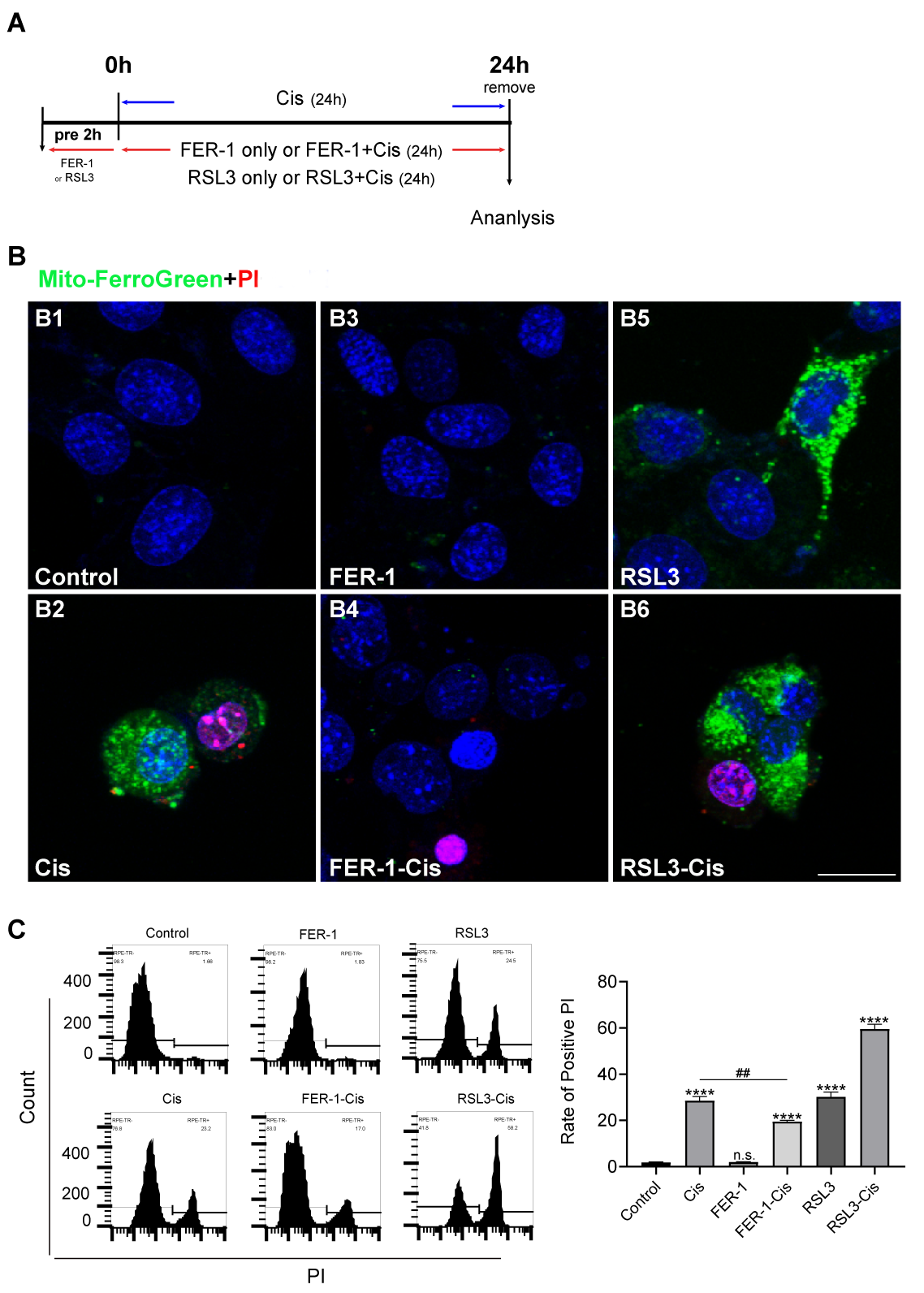


**Supplementary Figure 2**

Effect of FER-1 on cell death in cisplatin-damaged HEI-OC1 cells. (A) The experimental workflow for (B-C). The HEI-OC1 cells were pre-treated with 30 μM FER-1 or 3 μM RSL3 for 2 h and then treated with or without 30 μM cisplatin for another 24 h, or treated with 30 μM cisplatin alone for 24 h, and then cell death was detected by propidium iodide (PI). (B) Representative images of Mito-FerroGreen (green) in HEI-OC1 cells co-stained with PI (red). Nucleus was stained with Hoechst 33342 (blue). Scale bar, 20 µm. (C) PI was calculated according to the result of flow cytometric analysis. The data is shown as mean ± SEM. of three independent experiments. *****p* < 0.0001 and n.s. no significant vs. the control group; ^##^*p* < 0.01 vs. the cisplatin group.

**
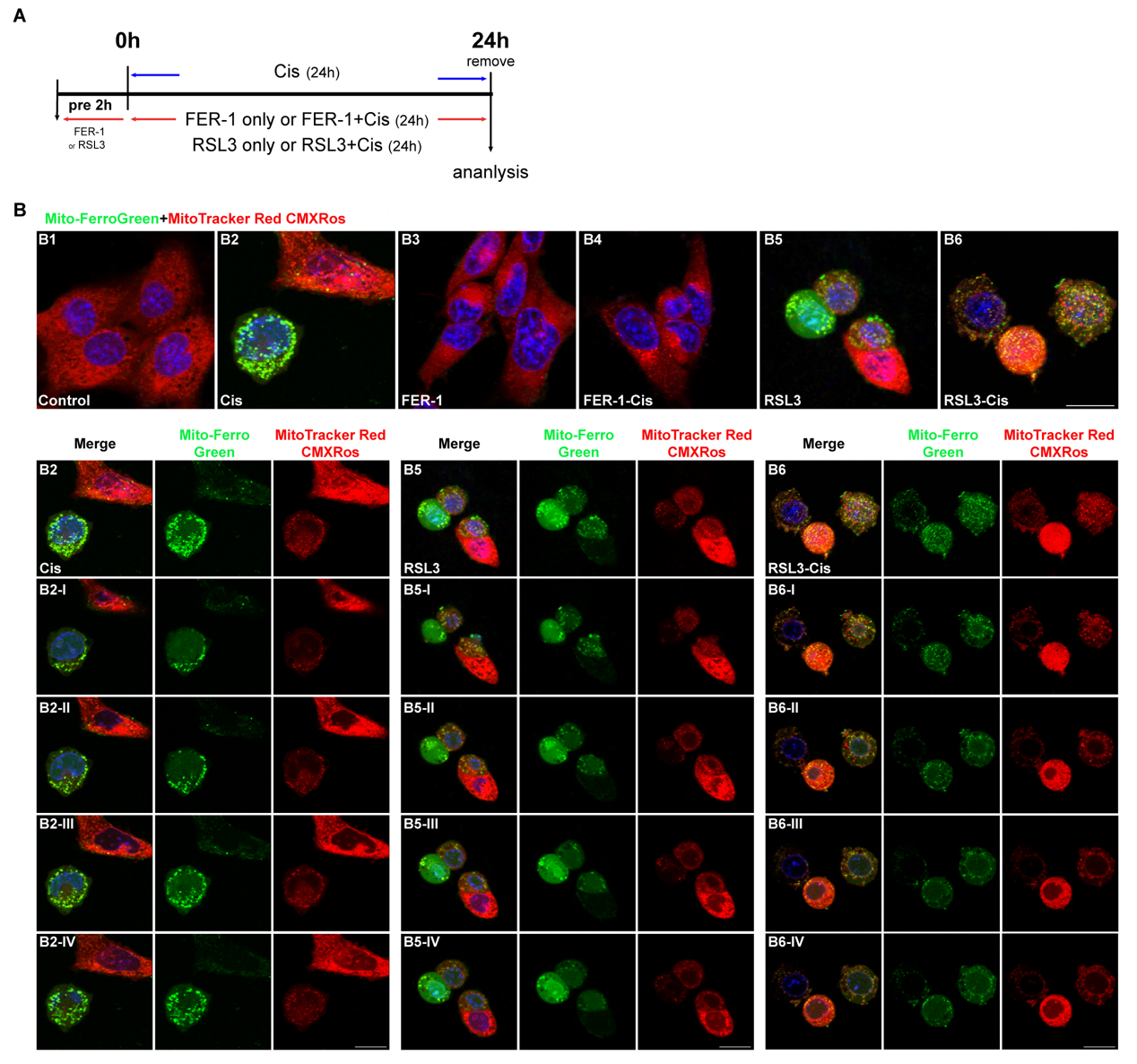
**

**Supplementary Figure 3**

Effect of FER-1 on mitochondrial morphology in cisplatin-damaged HEI-OC1 cells. (A) The experimental workflow. The HEI-OC1 cells were pre-treated with 30 μM FER-1 or 3 μM RSL3 for 2 h and then treated with or without 30 μM cisplatin for another 24 h, or treated with 30 μM cisplatin alone for 24 h. (B) Co-localization analysis of Mito-FerroGreen (green) with MitoTracker Red CMXRos dye (red). Nucleus was stained with Hoechst 33342 (blue). Scale bar, 20 µm. B1-B6: a maximum projection of all slices in the stack; B2-(I-IV), B5-(I-IV), B6-(I-IV): a single confocal z-stack slice (depth = 1.5 µm). The images shown here are representative of three independent replicate experiments.


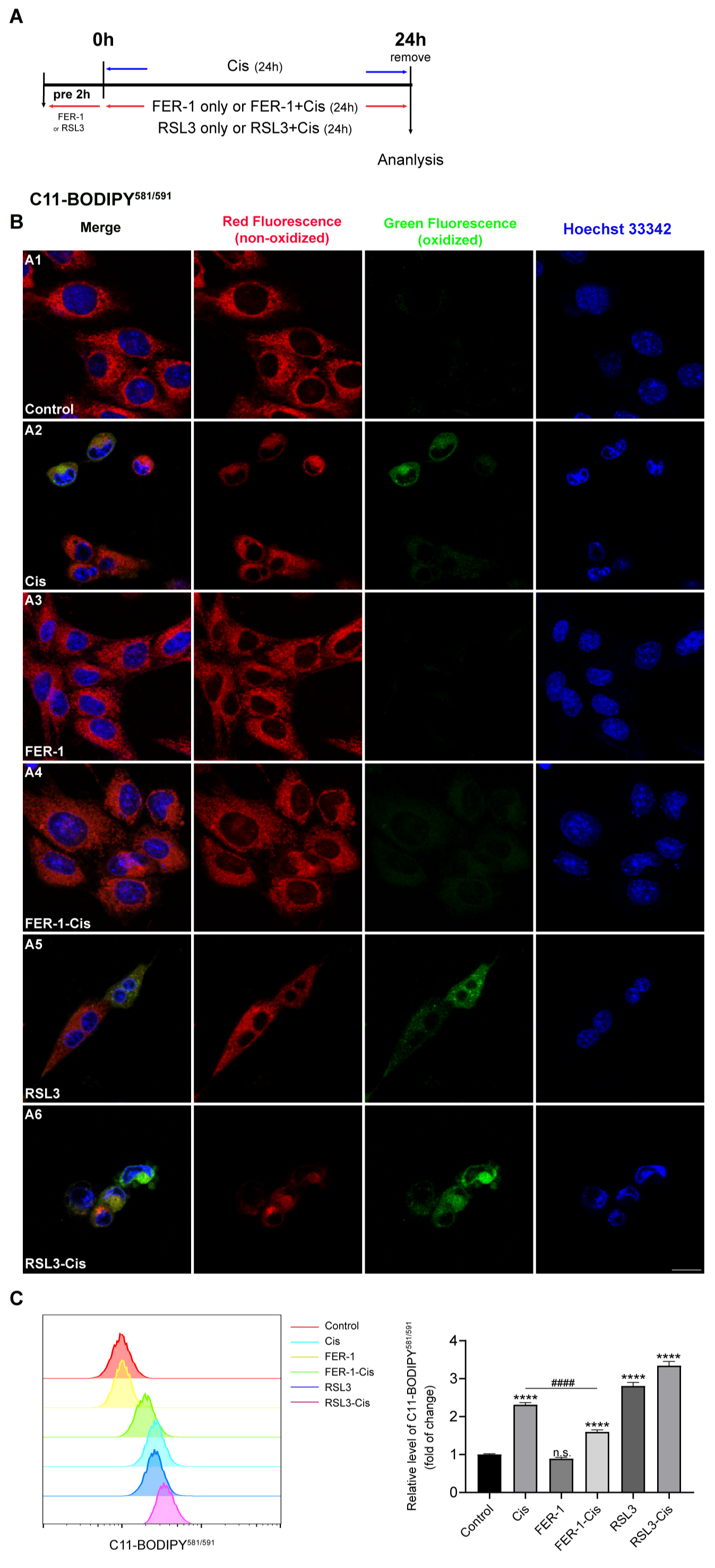


**Supplementary Figure 4**

Effect of FER-1 on lipid ROS production in cisplatin-damaged HEI-OC1 cells. (A) The experimental workflow for (B-C). The HEI-OC1 cells were pre-treated with 30 μM FER-1 or 3 μM RSL3 for 2 h and then treated with or without 30 μM cisplatin for another 24 h, or treated with 30 μM cisplatin alone for 24 h, and then lipid ROS production was detected by C11-BODIPY^581/591^ fluorescent probe. (B) Representative images of C11-BODIPY^581/591^ staining. The red is representative of non-oxidized lipid images of cells labeled with C11-BODIPY^581/591^ while green images represent oxidized lipid. Nucleus was stained with Hoechst 33342 (blue). Scale bar, 20 µm. (C) Quantification of C11-BODIPY^581/591^ by flow cytometry. The data is shown as mean ± SEM. of three independent experiments. *****p* < 0.0001 and n.s. no significant vs. the control group; ^####^*p* < 0.0001 vs. the cisplatin group.


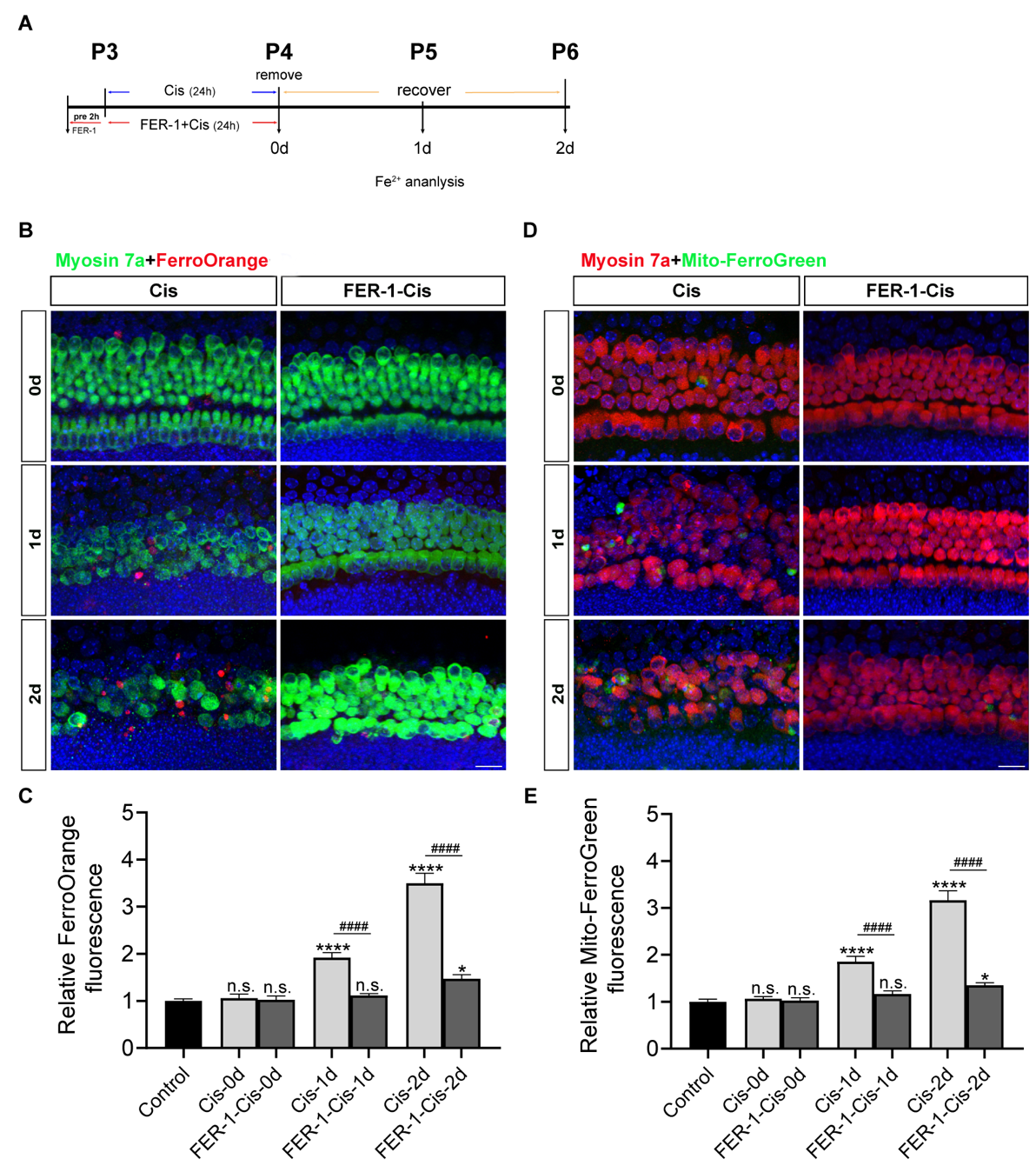


**Supplementary Figure 5**

Effect of FER-1 on iron production in cisplatin-damaged cochlear hair cells. (A) The experimental workflow for (B-E). Cochlear explants were treated with 30 μM cisplatin alone (Cis) or pretreatment with 30 μM FER-1 for 2 h and addition of 30 μM cisplatin for 24 h followed by 0, 1, and 2 days recovery. (B) Representative images of myosin 7a (green) and FerroOrange (red) staining of middle cochlear turns from different groups. Scale bar, 20 µm. (C) Relative fluorescence intensity of FerroOrange. (D) Representative images of myosin 7a (red) and Mito-FerroGreen (green) staining of middle cochlear turns from different groups. Scale bar, 20 µm. (E) Relative fluorescence intensity of Mito-FerroGreen. The columns indicate the mean ± SEM. **p* < 0.05, *****p* < 0.0001 and n.s. no significant vs. the control group; ^####^*p* < 0.0001 vs. the cisplatin group, n = 9-15 cochlear explants from three independent experiments.


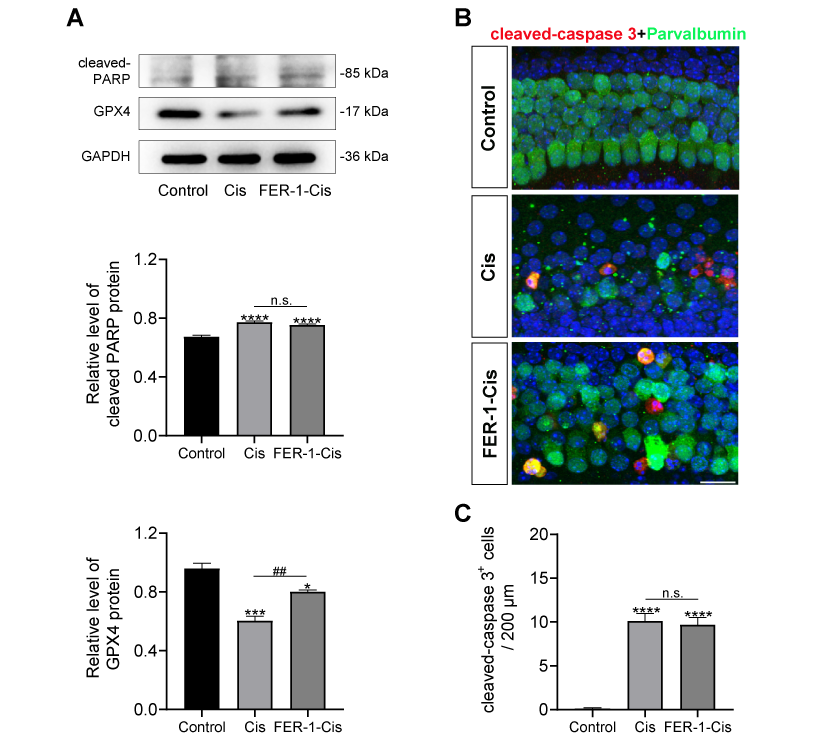


**Supplementary Figure 6**

Effect of FER-1 on cell apoptosis in cisplatin-damaged cochlear explants. Cochlear explants were treated with 30 μM cisplatin for 24 h (Cis) or pre-treated with 30 μM FER-1 for 2 h followed by co-treatment with 30 μM cisplatin for 24 h (FER-1-Cis). (A) Western blot analysis of GPX4 and cleaved-PARP. Quantification of levels of GPX4 and cleaved-PARP protein assessed by immunoblot. The data is shown as mean ± SEM. of three independent experiments. **p* < 0.05, ****p* < 0.001, *****p* < 0.0001 vs. the control; ^##^*p* < 0.01 and n.s. no significant vs. the group treated with cisplatin alone. (B) Representative images of parvalbumin (green) and cleaved-caspase 3 (red) staining of middle cochlear turns from different groups. Scale bar, 20 µm. (C) Quantification of cleaved-caspase 3-positive cells in the middle turns of different groups. The data is shown as mean ± SEM. *****p* < 0.0001 vs. the control group; n.s. no significant, n = 12 cochlear explants from three independent experiments.
